# Potential effects of gut microbiota on host cancers: focus on immunity, DNA damage, cellular pathways, and anticancer therapy

**DOI:** 10.1038/s41396-023-01483-0

**Published:** 2023-08-08

**Authors:** Jiaao Sun, Feng Chen, Guangzhen Wu

**Affiliations:** Department of Urology, The First Affiliated Hospital of Dalian Medical University, Dalian, 116011 China

**Keywords:** Bacteriology, Pathogenesis, Diseases

## Abstract

The symbiotic bacteria that live in the human gut and the metabolites they produce have long influenced local and systemic physiological and pathological processes of the host. The gut microbiota are increasingly being recognized for its impact on a range of human diseases, including cancer, it may play a key role in the occurrence, progression, treatment, and prognosis of many types of cancer. Understanding the functional role of the gut microbiota in cancer is crucial for the development of the era of personalized medicine. Here, we review recent advances in research and summarize the important associations and clear experimental evidence for the role of the gut microbiota in a variety of human cancers, focus on the application and possible challenges associated with the gut microbiota in antitumor therapy. In conclusion, our research demonstrated the multifaceted mechanisms of gut microbiota affecting human cancer and provides directions and ideas for future clinical research.

## Introduction

In recent years, a large amount of evidence has shown that parasitic microorganisms in the human body are key factors in health or pathological conditions. Diseases including inflammatory bowel disease, atherosclerosis, multiple sclerosis, diabetes, and Alzheimer’s disease, among others are associated with dysbacteriosis [1, 2]. With the increase in the incidence of malignant tumors, the interactions between microbiota and cancer are increasingly emerging.

The historical record linking cancer and microbes dates back to 1868, when William Busch reported spontaneous tumor regression in cancer patients infected with Streptococcus pyogenes. Among the human microorganisms, the gut microbiota are the most widely studied; approximately 3 × 10^3^ types of microbial cells inhabit the human body, numbering up to 4 × 10^13^ organisms in total, of which approximately 97% are gut microbiota. *Firmicutes*, *Bacteroidetes*, *Actinomycetes*, *Proteobacteria*, and *Verrucomicrobia* are the main bacterial groups present in the gut microbiota [3–6].

In 2015, Guinney et al. classified colorectal cancer (CRC) based on genes differentially expressed in tumor cells, resulting in a powerful classification system for CRC, the consensus molecular subtypes (CMS). These include CMS1 (immunoinvasive type, 14%), CMS2 (classical type, 37%), CMS3 (metabolic dysregulation type, 13%), and CMS4 (stromal invasion type, 23%) [7]. Later, Purcell et al. found that different CMS subtypes were associated with different gut microbiota compositions. Using 16 S rRNA gene sequencing, they found enrichment of *Fusobacteria* (15.7%) and *Bacteroidetes* (48.5%), and the absence of *Firmicutes* (<3%) and *Proteobacteria* (<3%) in CMS1 patients (the expression of immunoinfiltration-related genes is significantly increased) [8]. At the same time, CMS2 patients showed enrichment of *Selenomas* and *Prevotella spp*. (genes significantly associated with cell cycle were significantly upregulated in CMS2 patients). [7, 8]. This suggests that the composition of gut microbiota affects the immune and genetic patterns of CRC and other cancers, and the mechanism may be complex.

In recent years, studies in metabolomics and genomics have emphasized the dual role of gut microbiota in cancer prevention, occurrence and anticancer therapy; that is, the gut microbiota can both suppress and promote tumors [9]. In this review, we discuss how the gut microbiota play a role in human cancer and its application in anticancer therapy.

## Gut microbiota play a role in the development and progression of cancer

Experimental models have shown that as the largest microbiota in human body, gut microbiota play a key role in cancer by influencing immunity, genetic material, and cell pathways [10] (Supplementary information: Supplementary Table).

In recent years, 16 S rRNA gene sequencing and metagenomic analysis have revealed widespread differences in gut microbiota diversity between cancer patients and healthy individuals [11]. 16 S rRNA is an rRNA involved in the manufacture of small subunits of prokaryotic ribosomes; it provides low-cost and reliable identification of the overall microbiome composition [12, 13]. Unlike 16 S rRNA gene sequencing, metagenomic analysis does not target a specific microbial population, nor does it sequence a single microbial population. Instead, metagenomic analysis is performed as a sum of all microbial genomes. As Laudadio et al report, metagenomic sequencing analysis can help characterize the complexity of the microbiome in greater detail than 16 S rRNA gene sequencing [14]. The relationship between the gut microbiota and cancer continues to be elucidated owing to the use of increasingly advanced microbial detection techniques.

Below, we explore the role of gut microbiota in cancer through different mechanisms, including immunity, DNA damage, cellular signaling pathways, and inflammasomes (Table 1).

**Table 1:** Effects of a single gut microbiota on the development of multiple cancers.

GM	Promote/Inhibit	Cancer types	Model	Commentary	References
***F. Nucleatum***	Promote	CRC	Human colorectal cancer cell line RKO	Secretes Fap2, binds to and blocks NK receptors, recruits MDSCs, and indirectly promotes cancer	[25]
	Promote	CRC	Apc(Min/+) mouse model	Recruit tumor-infiltrating immune cells to create an inflammatory microenvironment conducive to colorectal tumor progression	[26]
	Promote	CRC	Human colorectal cancer cell line HCT116, DLD1, SW480 and HT29	FadA is secreted, which binds to the 11-aa region on E-cadherin, inhibits E-cadherin activity and drives CRC genesis	[150]
	Promote	Esophageal squamous cell carcinoma	TE-8 and TE-10 cell lines	Promote the progression of esophageal squamous cell carcinoma by NOD1/RIPK2/NF-κB pathway	[151]
	Promote	Pancreatic cancer	BxPC3, Panc1, HPAC, and Capan1 cell lines	Induce the secretion of cytokines GM-CSF, CXCL1, IL-8 and MIP-3α to promote the proliferation of cancer cells	[152]
***B. fragilis***	Promote	CRC	Human colorectal cancer cell line HT29/C1	Secretes BFT, activates NF-kappaB and MAPKs, promotes TH17 response, secretes IL-8, significantly increases the formation of colon tumors	[39]
	Inhibit	CC	CC patients, mouse model	Induce apoptosis of ileal crypt intestinal epithelial cells and recruit TFH, which interferes with proximal colonic tumors in IL-1R and IL-12-dependent manners and inhibits tumor growth	[49]
	Promote	CRC	Human colorectal cancer cell line HT29/c1, T84	Upregulation of SMO leads to the production of SMO-dependent ROS and the induction of γ-H2A.X, which further leads to DNA damage and induces carcinogenesis	[58]
	Promote	CRC	Human colorectal cancer cell line HT29, T84	Induces the expression and basal secretion of CXC chemokine, thereby stimulating the expression and production of IL-8 and growth-associated oncogene-α (GRO-α).	[153]
***Bifidobacterium***	Inhibit	Melanoma	C57BL/6 mouse model	Enhance DC function, activate CD8 + T cell response in tumor microenvironment, and improve anti-tumor immune effect	[45]
	Inhibit	CRC	CRC mouse model	Inosine metabolites secreted and perinosine-A2aR-cAMP-PKA pathway induce Th1 differentiation and effector function, which is beneficial for anti-tumor immune processes	[42]
***A. muciniphila***	Inhibit	CRC	CAC mouse model	Secreting villiform TLR2 agonists induces TNF-α production from CTLs in mesenteric lymph nodes, alleviating the incidence of colitis-related colon cancer	[154]
***Lactobacillus casei***	Inhibit	Skin cancer	BALB/c mouse model	Stimulates DC cells to secrete IL-12 and inhibits tumor formation	[155]
	Inhibit	CRC	CRC mouse model	Stimulates the regulation of the immune response of Treg cells to biased TH17, accompanied by the expression of regulatory cytokines IL-6, IL-17, IL-10 and TGF-β	[156]
***Staphylococcus***	Inhibit	SpC	C3H/HeN mouse model (Subcutaneous injection of squamous cell carcinoma SCC VII cell line)	The secretion of SEB significantly increased the expression levels of STAT5 and HDAC-1 in CD4 + T cells, resulting in increased IL-9 secretion and inducing apoptosis in SqC cells	[51]
***E. coli***	Promote	CRC	Human colorectal cancer cell line (Caco-2), small intestine untransformed cells (IEC-6)	Secretes cell cycle inhibitors that interfere with host regulation of the normal cell cycle	[157]
	Promote	CRC、Cervical cancer	HeLa cell line, C57BL/6 J mouse model	Colibactin alkylates DNA on adenine residues and induces double-strand breaks, resulting in mutations that can directly lead to colon cancer	[52]
	Promote	CRC	Human colorectal cancer cell line HT29 and SW480	Secretes EspF, targets the mitochondria of intestinal epithelial cells, and induces post-translational modification of mismatch repair proteins and their degradation	[66]
	Promote	CRC	Mouse intestinal loop model, CHO cell line	The genomic islands of its "PKS" encode the production of genotoxins, resulting in phosphorylated histone H2AX lesions that induce the development of sporadic colorectal cancer	[158]
***H. pylori***	Promote	CRC	C57BL/6 mouse model	Activates the host’s SMO, produces cells with oxidative DNA damage and anti-apoptotic ability, and induces carcinogenesis	[57]
	Promote	Stomach cancer	Human gastric adenocarcinoma cell line AGS	Interferes with the AKT pathway of host cells, induces proteasome-mediated degradation of p53 in gastric epithelial cells	[62]
	Promote	Stomach cancer	Human gastric cancer highly metastatic cell line MKN28	CagA protein secreted interacts with E-cadherin secreted by host epithelial cells, destroys intercellular junctions, activates β-catenin signaling, and increases the risk of host cell carcinogenesis	[159]
	Promote	CRC	Mouse model(GPX knocked out)	Inhibits the function of GPX-1 and GPX-2, improves the cellular oxidative environment, and increases the possibility of carcinogenesis of ileal and colonic epithelial cells	[160]
***Enterococcus faecalis***	Promote	CRC	Wistar rat model	The NOX2-dependent production of extracellular superoxide and derived oxygen increases the potential for DNA mutations in host cells	[56]
	Promote	CRC	Wistar rat model	Through cell membrane-related oxidation, large amounts of extracellular peroxides and reactive oxygen species are produced, resulting in colorectal cancer-related CIN	[161]
***Shigella flexner***	Promote	CRC	HeLa cell line, HCT116 cell line	Secretion of IpgD and VirA induces p53 degradation in host cells, increasing the likelihood of DNA mutations	[63]
***Morganella morganii***	Promote	CRC	HeLa cell line, CRC mouse model	Produces DNA toxic metabolite, indoleamine, increases DNA mutation rate and intestinal permeability	[65]
***Butyrivibrio fibrisolvens***	Inhibit	CRC	BALB/c GF mouse model	Secretes butyrate, inhibits histone deacetylase activity, increases the degree of acetylation of intracellular histones, inhibits the proliferation of tumor cells	[162]
***Porphyromonas gingivalis***	Promote	CRC	Human colorectal cancer cell line (LS174T), mouse colon cancer cell line (MC38)	Gingival protease secretes, activates the MAPK/ERK signaling pathway to invade host cells, and promotes the proliferation of colorectal cancer cells	[163]
***Bacteroides polymorpha***	Inhibit	CRC	Mouse colon cancer cell line CT26、CRC mouse model	The OMV secreted by it re-edited TME across the intestinal epithelial barrier, allowing Th0 cells to differentiate towards TH1 type to secrete CXCL10 and IFN-, which has a cancer-suppressing effect	[164]
***L. casei***	Inhibit	CRC	Human colorectal cancer cell line Caco2, SKCO-1, SW620	Its derived iron chromate is directly activated through the JNK pathway to trigger apoptosis of tumor cells	[165]
	Inhibit	Cervical cancer	HeLa cell line	up-regulate the expression of apoptotic genes BAX and BAD, and down-regulate the expression of BCl-2	[166]
***Leuconostoc mesenteroides***	Inhibit	CRC	Human colorectal cancer cell line HT-29	Up-regulation of the expression of MAPK1, Bax and caspase 3, and downregulation of key factors such as AKT, NF-kB, Bcl-XL, miRNA-21 and miRNA-200b, significantly promoted the apoptosis of colon cancer cell lines	[167]
***B.rodentium***	Inhibit	Melanoma	Rnf5 − /− C57BL/6 mouse model(subcutaneous injection of melanoma cell lines)	Increase the local recruitment of DC cells and enhance anti-tumor immunity	[47]
***Lactobacillus rhamnosus GG***	Inhibit	Hepatocellular carcinoma	HepG2 cells line	Use extracellular vesicles, it has significant cytotoxic effect on cancer cells	[168]
	Inhibit	Oral cancer	HSC-3 cell line	Enhance the anticancer effect of gardenoside in HSC-3 cells	[169]
	Inhibit	CRC	HT29 cell line	Relieve oxidative stress induced by hydrogen peroxide	[170]

### Immune microenvironment

#### Gut microbiota and antitumor immunity

**Fig. 1:**
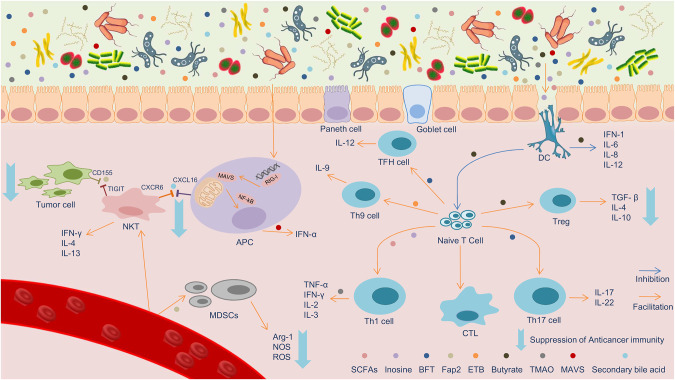
Gut microbiota metabolites are involved in innate and adaptive host immunity. The gut microbiota and metabolites in the intestinal lumen are sensed by dendritic cells (DCs), which then induce the transformation of naive T-cells into various effector T-cells. In this process, butyrate inhibits DC activation of naive T-cells and secretion of IL-6, −8, and −12, and other factors, while promoting the transformation of naive T-cells into Treg. *Bacteroides fragilis* toxin (BFT) promoted the transformation of naive T-cells into Tfh cells and Th17. Staphylococcal enterotoxin B promotes the transformation of naive T-cells into Th9 cells. Inosine and short-chain fatty acids promote the transformation of naive T-cells into Th1 cells, and trimethylamine N-oxide promotes the secretion of IL-2 and −3, and other factors by Th1 cells. In terms of innate immunity, myeloid-derived suppressor cells reach the intestinal tract through the blood and secrete cancer-promoting factors, such as Arg-1, nitric oxide synthase (NOS), and reactive oxygen species. In this process, FAP2 plays a promoting role. Simultaneously, FAP2 binds and blocks the receptor TIGIT on NK T-cells, thus inhibiting the NK T-mediated tumor cell attack process. Secondary bile acid decreases the expression of CXCL16 on the surface of antigen-presenting cells (APCs) and prevent the aggregation of CXCR6^+^ NK T-cells. Intracellular RIG-I on APCs recognizes the abnormal DNA of the bacterial community and transmits the signal to mitochondrial anti-viral signaling proteins (MAVS) on the mitochondrial membrane, which in turn activates the NF-kB signal and releases IFN-α, a process in which MAVS plays a catalytic role.

In recent years, more and more studies have focused on the regulatory effect of microflora on host immunity. Researchers often associate tumor immunology with microbiota [15], especially non-pathogenic microbiota, which is often not the direct cause of some diseases, but affects local and distant pathological processes, among which gut microbiota has a complex and critical interaction mechanism with the immune system. The gut microbiota helps the immune system tolerate foreign antigens from food; furthermore, it helps the immune system recognize and eliminate pathogenic bacteria, thereby preventing the invasion of pathogens [16]. In general, gut microbes and products produced by microbes (such as short-chain fatty acids [SCFAs]) activate immune responses by interacting with immune cells expressing toll-like receptors (TLRs). For example, SCFAs will promote the differentiation of naïve T-cells to Th1 cells, thereby enhancing immunity, and dendritic cells (DCs) activated by SCFAs migrates from the GI tract to the mesenteric lymph nodes, inducing naïve T-cells to transform into effector T-cells, different types of effector T-cells migrate to the gastrointestinal tract again and stimulate local immune responses, while the remaining cells enter the systemic circulation, affecting system-wide immunity [17–22]. In short, the gut microbiota interact with immune cells or alters the immunogenicity of tumor cells, affecting the antitumor effect of the host immune system and even leading to differential responses to immunotherapy [23]. These complex and important regulatory processes are described in detail below (Fig. 1).

#### Gut microbiota and innate immunity

Physiological protective barrier is one of the important components of innate immunity, including intestinal and symbiotic bacteria. It has been reported that the abundance of *Lactobacillus* and *Bifidobacteria* in breast milk plays an important role in the construction of neonatal innate immunity [24]. During innate immunity, *Fusobacterium nucleatum* can inhibit host natural killer (NK) cells and recruit myeloid-derived suppressor cells (MDSCs) at the site of infection, thereby indirectly promoting the occurrence of cancer. This process is mediated by the bacterial virulence factor FAP2, which binds to and blocks the NK receptor TIGIT, thereby inhibiting the NK-mediated immune system’s attack on tumor cells [25]. Similarly, in a mouse model of CRC, *Fusobacterium* inhibited T-cell responses by recruiting tumor-infiltrating immune cells and manipulating the innate immune system, producing an immune microenvironment conducive to colorectal tumor progression [26]. Secondary bile acids produced by gut microbiota decreased CXCL16 (the only ligand for CXCR6 (CXC Receptor 6)) expression on hepatocyte surface, demonstrated by higher levels of CXCL16 mRNA in the liver of germ-free mice (about twice), which prevent the aggregation and immune monitoring of CXCR6^+^ T-cells, thereby causing liver cancer. This immune escape reaction can be eliminated by antibiotic treatment such as vancomycin [27].

#### Gut microbiota and primary lymphoid organs

The gut microbiota also induce TNF-α expression via tumor-associated natural bone marrow cells, mediating TNF-dependent early tumor necrosis [28]. In addition, after bone marrow transplantation, a decrease in the number of gut microbiota count may exacerbate systemic infection and increase the radiation sensitivity, whereas higher gut microbiota diversity significantly improved the efficacy of allogeneic hematopoietic stem cell transplantation in leukemia patients. Specifically, there were 104 deaths among 354 patients in the high-diversity group and 136 deaths among 350 patients in the low-diversity group [29]. Subsequent studies have shown that certain compounds produced by gut microbial metabolism, such as propionic acid and tryptophan, enhance the function of bone marrow cells and neutrophils derived from bone marrow transplantation and prevent hematopoietic injury caused by bone marrow transplantation [30–32]. Translocation of gut bacteria in mice also exacerbates pre-leukemia bone marrow dysplasia, which can lead to precursor B-cell acute lymphoblastic leukemia (pB-ALL). Of a total of 23 mice with pB-ALL defects, 11 developed pB-ALL between 11 and 20 months of age, while none developed in germ-free mice (*n* = 12) [33]. The effects of the gut microbiota on lymphoid organs may be due to the activation of mitochondrial anti-viral signaling proteins (MAVS) by endogenous ligands (such as viruses, bacteriophages, or bacteria-derived RNA) of RIG-I (a receptor that recognizes abnormal mRNA in cells), which in turn induces protective signaling by IFN-I [34].

#### Gut microbiota and adaptive immunity

In adaptive immunity, the gut microbiota elicit host-specific T-cell responses in an antigen-presenting manner, in which the microbiota and its metabolites significantly affect the body’s antitumor immune effects [35, 36]. T-cell differentiation can be divided into three effector pathways: Th1, Th2, and Th17 responses. The STAT1 and STAT4 signaling pathways promote the Th1 response, the STAT6 signaling pathway promotes the Th2 response, and the STAT3 signaling pathway promotes the Th17 response. The Th1 response is characterized by the production of IFN-γ, which generally has anticancer effects, although it also plays roles in allergic and inflammatory reactions. However, the toxin secreted by *Bacteroides fragilis* (BFT) significantly increases colon tumor formation by rapidly, strongly, and selectively activating STAT3 and promoting the Th17 response, a process accompanied by the activation of serine/threonine mitogen-activated protein kinase (MAPK) and NF-kB signaling [37–40]. In addition, Trimethylamine N-oxide (TMAO), a metabolic derivative produced by gut microbiota that helps the body metabolize choline or trimethylamine foods, has been demonstrated to enhance the antitumor immunity to pancreatic ductal adenocarcinoma in mouse models; researchers have described the mechanism by which TMAO enhances the INF-I signaling pathway and enhances the antitumor effect in an INF-I-dependent manner [41]. Inosine is a purine metabolite that acts as an important modulator of the immune checkpoint blockade therapy response. *Bifidobacterium dentium pseudotudes* and *Lactobacillus johnsonii* in the intestine produce inosine in the systemic circulation and induce Th1 differentiation and effector function [42].

#### Gut microbiota and immune cells

DCs are among the most important antigen-presenting cells in the human body, antigens produced by gut microbiota or their metabolites can be used to activate dc to reverse immune tolerance induced by immature DCs [43]. DCs from the gut-associated lymphoid tissue area sense various gut microbiota antigens, including *Bifidobacteria*, *Bacteroides fragilis*, *Myxobacterium*, *Bacillus rodentia*, *Bacteroides*, and their metabolites, and catalyze immune reactions through IFN-I and IL-12 [44–47]. In a melanoma mouse model, *Bifidobacteria* activated DCs through the TLR4-mediated signaling pathway, and then DCs amplified the CD8^+^ T-cell response in the tumor microenvironment. Through ELISPOT and flow cytometry, it was found that the mechanism was to strongly induce peripheral tumor-specific T cells and to increase the accumulation of antigen-specific CD8 + T cells in the tumor [45]. Conversely, it has also been reported that elevated levels of gut microbiota metabolites, such as butyric acid and propionic acid, increase the proportion of Treg cells and decrease DC activation, which, in turn, leads to reduced effector T-cells and IL-2, and even tolerance to cytotoxic T lymphocyte-associated antigen 4 (CTLA-4) blockers [48].

Additionally, Tfh cells are an important member of the adaptive immune family, and are present in mucosal lymphoid tissue and tumour-draining lymph nodes. Apoptosis of ileal crypt intestinal epithelial cells can induce Tfh cells to interfere with proximal colon tumors in an IL-12-dependent manner, inhibit the growth of tumor cells. This immune response depends on the microbiome of the ileum site, such as *Bacteroides fragilis* [49]. Th9 cells are important immune cells that secrete IL-9 in the intestinal lamina propria. If the expression of IL-4 and TGF-β and the number of Th9 cells in germ-free mice decrease, the probability of melanoma growth increases. After transplantation into germ-free mice, IL-9 production was restored and tumor growth was reduced [50]. Another study showed that Th9 cells exposed to staphylococcal enterotoxin B (ETB) significantly promoted apoptosis in tumor cells. As an antigen, ETB significantly increased the expression levels of STAT5 and HDAC-1 in CD4^+^ T-cells, resulting in increased IL-9 secretion [51].

### DNA damage

**Fig. 2:**
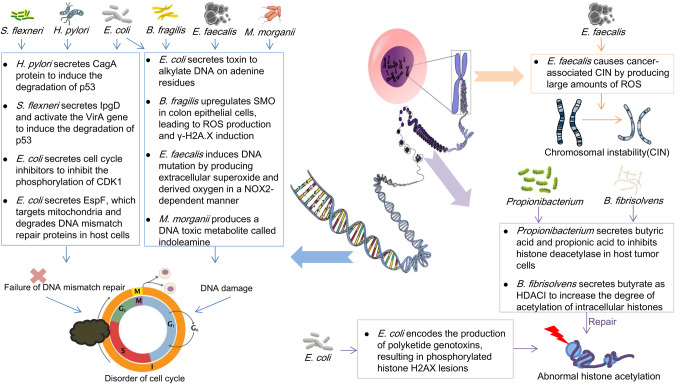
DNA mismatch-repair imbalance, DNA damage, chromosomal instability, and abnormal histone acetylation caused by gut microbiota. Gut microbiota such as *Shigella flexneri* (*S. flexneri*), *Escherichia coli* (*E. coli*), *Bacteroides fragilis* (*B. fragilis*), *Enterococcus faecalis* (*E. faecalis*), *Morganella morganii* (*M. morganii*), and *Helicobacter pylori* (*H. pylori*) block the normal cell cycle by affecting oxidative environment-dependent DNA damage and disrupting the DNA mismatch-repair process, thus increasing the tendency of epithelial cells to become cancerous. Additionally, toxins secreted by *E. coli* interfere with histone acetylation, while butyrate and propionic acid, metabolites of *Butyrivibrio fibrisolvens* (*B. fibrisolvens*) and *Propionibacterium*, as inhibitors of deacetylase, can increase the degree of acetylation and have an opposite anticancer effect. *E. faecalis* releases oxidants through a macrophage-dependent manner, causing chromosomal instability.

Genotoxins released by gut microbiota exhibit DNase activity. Once released near gastrointestinal epithelial cells, these toxins cause double-stranded DNA to break in host epithelial cells, resulting in a brief arrest of the cell cycle. The first known mutagenic effect was observed in *Escherichia coli*. Colibactin from *Escherichia coli* can induce double-strand breaks by alkylating adenine residues in DNA, leading to direct mutations that can trigger CRC [52]. Since then, various studies have found that colibactin, cell-lethal bulking toxin, and BFT cause genetic mutations to varying degrees in colorectal, head and neck, urothelial, and other cancers [52–55] (Fig. 2).

Oxidation of the environment is an important cause of DNA damage. For example, analysis of ESR (electron spin resonance) spectra after *Enterococcus faecalis* colonization showed that *E. faecalis* produced extracellular superoxide and derived oxygen in a NOX2-dependent manner, which oxidizes the environment when diffused into the host cell and increases the likelihood of DNA mutations in the host cell [56]. Reactive oxygen species (ROS) production may be reduced by antibiotic use or intestinal sterility [28]. Similarly, *Helicobacter pylori* activate spermine oxidase in the host, producing large amounts of hydrogen peroxide and reactive oxygen species, inducing DNA mutation and carcinogenesis [57, 58]. SMO (spermine oxidase) is a metabolic enzyme induced by inflammatory signals. In colon cancer cells, BFT rapidly induced SMO gene expression, resulting in a 2- to 4-fold increase after 3 or 6 h of exposure, respectively, resulting in the production of SMO-dependent ROS and dysregulation of Gamma-H2AX, which further leads to DNA damage and induces carcinogenesis. Gamma-H2AX is the phosphorylated form of H2AX, involved in DNA repair when DNA breaks and cell cycle abnormalities occur [58] (Fig. 2).

As a tumor suppressive transcription factor, p53 can bind to specific DNA sequences and activate transcription, regulate unbalanced cell cycle, and repair defective genes. Common oncogenic p53 mutations usually occur when mediated by metabolites produced by the gut microbiota [59]. The protein CagA, produced by *Helicobacter pylori*, was the first bacterial protein to be shown to be associated with human cancer, impairing the repair process of DNA mismatches in gastrointestinal epithelial cells [60, 61]. CagA can interfere with the AKT pathway in host cells thereby promoting the occurrence of gastric cancer, after HCT116 cells were infected with the specified *H. pylori* strain for 10 h, western blot analysis showed a large degree of p53 degradation [62]. Similarly, *Shigella flexneri* also induces host cell p53 degradation through the secretases IpgD and VirA, increases the frequency of DNA mutations [63] (Fig. 2).

In addition, a large number of similar studies have assessed the ability of the gut microbiota to influence host DNA integrity. For example, a clinical study found that highly pathogenic mutations in the APC tumor suppressor gene in the intestinal cells of patients were associated with an increase in *Fusobacterium mortiferum* and a significant decrease in *Clostridium geniculate* and *Bifidobacteria* [64]. *Morganella morganii* produces a novel DNA toxic metabolite, indoleamine, which increases DNA mutation rate and intestinal permeability in mouse models of colon cancer and increases tumor load [65]. In SW480 cells transfected with a fusion gene containing 12 continuous cytosine residues, a significant increase in infection-induced cell point mutations of EPEC (Enteropathogenic *E.coli*) was observed compared to those for infection. Mechanistically, EPEC consumes host cell DNA mismatch-repair proteins by secreting EspF, which specifically targets the mitochondria of intestinal epithelial cells to induce their degradation [66] (Fig. 2).

In addition to DNA damage and variation, gut microbiota is also associated with chromosome stability, epigenome change, and microRNA, which we have detailed in the supplementary information (Supplementary information: supplement to the article).

### Tumor-related signaling pathways

**Fig. 3:**
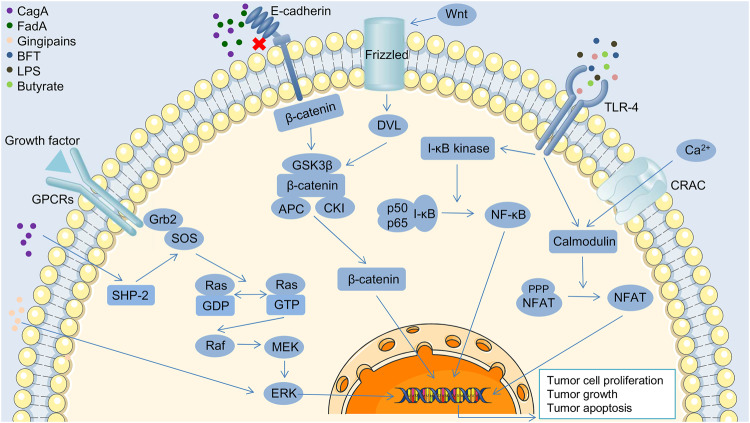
Gut microbiota are involved in the regulation of several intracellular signaling pathways. CagA binds to SHP-2 and activates it to promote Ras/MAPK signaling and trigger the abnormal proliferation of host T-cells. Gingipains is the main virulence factor of *Porphyromonas gingival*, it up-regulate phosphorylation of MEK and ERK, the core components of the RAS/RAF/MEK/ERK pathway, promoting cancer cell proliferation. E-cadherin is a transmembrane glycoprotein that exists in cell membranes and binds epithelial cells together to maintain their normal morphology and polarity. Normally, the intracellular peptide of E-cadherin is linked to β-catenin to ensure that it does not transmit signals to the nucleus, while the intracellular free β-catenin exists in the form of a complex. CagA and FadA destroy the function of E-cadherin, affect the intercellular connection and the binding force of β-catenin, and switch on the WNT/β-catenin signal when the WNT signal activates the cell membrane receptor FRZ. TLR-4 recognizes bacterial metabolites, such as *Bacteroides fragilis* toxin (BFT), gingipains, lipopolysaccharides, and butyrate, and activates the NF-kB and NFAT signaling pathways to promote abnormal proliferation of cancer cells.

Sparassis latifolia polysaccharides (SLPs), secreted by *Sparassis latifolia* (a fungus of the genus *Sparassis*), has been shown to influence the progression of colorectal cancer in mice through multiple mechanisms, including inhibiting the infiltration of immune cells, reducing the expression of inflammatory cytokines, and improving the metabolic disorders of cancer cells [67]. Thus, in addition to participating in the immune response and DNA damage, communication between the gut microbiota and the host can occur through a variety of mechanisms, including receptor ligand-mediated signaling and regulation of intestinal epithelial barrier function, which often leads to cancer progression. In this section, we seek to explore the role of the gut microbiota in the signaling of tumor-related pathways, both promoting and inhibiting cancer (Fig. 3). In addition, the components and secreted products of intestinal flora, such as LPS and SCFAs, are also involved in the occurrence and development of tumors, which are discussed in the supplement (Supplementary information: supplement to the article).

### Inflammasomes

Inflammasomes are a class of multiprotein intracellular complexes expressed in immune and epithelial cells that induce cell death under pathological conditions such as inflammation and stress, and their disorders can lead to a variety of diseases, including autoimmune diseases and cancer. The outcome of inflammasome activation depends on a variety of factors, including its expression pattern and effector molecules. The gut microbiota may also influence the activation of specific inflammasomes [68, 69]. By activating the inflammasome, the gut microbiota interact with the immune and intestinal epithelial cells, producing results that can be both cancer-suppressing and cancer-proactive [70, 71]. In a state of homeostasis, intestinal commensal bacteria activate the NLRP3 inflammasome production of IL-18, regulating adaptive immunity, which is essential for maintaining the integrity of the intestinal barrier and preventing dysbacteriosis [72–75]. Experiments have shown that inflammasomes weaken the occurrence of colitis and colitis-related tumors in mouse models, the effector factor IL-18 plays a key role in this action [74, 76–78]. Conversely, by releasing IL-1β, inflammasome activation promotes the development of lung, skin, breast, and pancreatic cancers, a process in which the gut microbiota also play an important role [79–83].

In addition to immune, genetic, cellular pathways, and inflammatory mediators, the gut microbiota also influence the development of hormone-dependent cancers. Patients with prostate cancer (PCa) often develop aggressive castration-resistant PCa because of alternative sources of androgens. *Clostridium scindens* is a member of the gut microbiota that converts cortisol into 11β-hydroxyl androstenedione, a potent androgen precursor. Cortisol metabolites derived from *Clostridium* s*cindens*-conditioned medium promote the proliferation and migration of androgen-dependent PCa cells, which indicate that *Clostridium scindens* promotes PCa progression by activating androgen receptor signaling [84].

## Moderating effect of gut microbiota on anticancer therapy

The combination of microbial and anticancer therapy began as early as the 19th century, when the first attempt was made to inject inactivated Streptococcus into human tumor tissues to cure cancer [85]. Previous studies have shown that microbial preparations injected directly into tumor tissues or administered orally can have a direct cytotoxic effect on tumor cells or stimulate the body’s local antitumor immune response. [86–89]. Recent studies have found that traditional radiotherapy, chemotherapy, and immunotherapy can alter the gut microbiota of patients, and that the composition of the flora can profoundly affect the efficacy and side effects of these treatments, including cancer recurrence, drug resistance, and collateral damage to the body (Table 2), we have analyzed this part of the content in detail and presented it in the supplementary information (Supplementary information: supplement to the article). Probiotics, prebiotics, and fecal microbiota transplantation (FMT) have also achieved some clinical efficacy; we will explain each of them below.

**Table 2:** Effects of gut microbiota on anticancer therapies, their toxic side effects and patient prognosis.

Treatment		GM	Assistance/Interference	Cancer types (Model)	Commentary	References
**Radiotherapy**		*Gram-positive bacteria that secrete butyrate (Clostridia)*	Interference	Melanoma, lung cancer and cervical cancer mouse model	The use of vancomycin eliminates Gram-positive bacteria secreting butyrate, promotes cross-presentation of CD8 + T cells, and enhances RT-induced anti-tumor immune response	[43]
		*Lactobacillus rhamnosus GG (LGG)*	Assistance	C57BL/6 mouse model exposed to whole-body radiation	The TLR-2/MyD88 signaling mechanism relocates cox-2-expressing mesenchymal stem cells to the base of the crypt, protecting the gut from radiotherapy-induced cell damage	[92]
**Chemotherapy**	Cyclophosphamide	*Lactobacillus johnsonii*	Assistance	C57BL/6 colorectal cancer mice	It led to the transformation of naïve T cells to TH17, induced the effect of CD8 + T cells producing INF, and improved the chemotherapy efficacy of tumor-bearing mice	[171]
	Cisplatin	*Lactobacillus*	Assistance	Lewi lung cancer mice	Lactic acid bacteria regulate the expression of genes such as VEGFA, Bax and CDKN1B in tumors and enhance adaptive immune responses	[172]
	5-Fluorouracil	*Lactobacillus plantarum*	Assistance	Human colorectal cancer cell line HT-29, HCT- 116	Lactobacillus plantarum enhances caspase-3 activity, inhibits cancer cell survival, activates Wnt/β-catenin signaling, and reduces tumor volume	[173]
		*Lactobacillus casei YIT 9018*	Assistance	BALB/c mouse model	As a nonspecific immunostimulant, reduces the lethal toxicity of 5-FU	[174]
	Oxaliplatin	*Fusobacterium nucleatum*	Interference	Human colorectal cancer cell line SW480 和HT-29	Targeting TLR4 and MYD88 innate immune signaling pathways and specific microRNAs to activate autophagy pathways and induce drug resistance	[175]
	Gemcitabine	*Gammaproteobacteria*	Interference	BALB/c colorectal cancer mice	Secrete bacterial enzyme cytidine deaminase (CDDL), Metabolizing Gemcitabine from the active form 2′,2′-difluorodeoxycytidine to 2′,2′-difluorodeoxyuridine and inactivating it produces drug resistance	[176]
**Immunotherapy**	CTLA-4 inhibitor	*B. thetaiotaomicron, B. fragilis*	Assistance	MCA205 Fibro sarcoma mice	Enhances IL-12-dependent TH1 immune response	[44]
		*Firmicutes*	Interference	26 melanoma patients	T cell co-stimulatory factor has a higher induction effect on CD4 + T cells, increases the number of serum CD25, and aggravates toxic side effects	[177]
		*Akkermansia muciniphila*	Assistance	Lymphoma GF mouse model	Microbial-derived STING agonists plan the transition of mononuclear phagocytes in TME to immunostimulated monocytes and DCs	[178]
		*Bifidobacterium pseudolongum*	Assistance	CRC mouse model	Inosine secretion acts through the T cell-specific A2AR signaling pathway to promote TH1 cell activation	[42]
	PD-1 inhibitor	*Bifidobacterium*	Assistance	C57BL/6 mouse model	Recruit DCs to activate CD8 + T cell responses in the tumor microenvironment	[45]
		*Akkermansia muciniphila*	Assistance	MCA-205 sarcoma, RET melanoma mouse model	Increase the recruitment of CCR9 + CXCR3 + CD4 + T lymphocytes in tumor tissues to restore the efficacy of PD-1 blockers in an IL-12-dependent manner	[120]
		*Bifidobacterium longum, Enterococcus faecium*	Assistance	42 melanoma patients, GF mouse model	Recruit DC cells, increase TH1 response, reduce Treg cells, enhance T cell response, and improve the efficacy of anti-PD-1 therapy	[118]
		*Clostridium, ruminococcus, or faecalis*	Assistance	112 melanoma patients	Increases antigen presentation-mediated systemic anti-tumor immune response, improves effector T cell function in peripheral and tumor microenvironment	[119]
	CD47 inhibitor	*Bifidobacterium*	Assistance	Bearing cancer mouse model	Stimulates the STING signaling pathway to increase the cross-primer of DC	[46]

### Probiotics

Probiotics are a class of bacteria that exist in the host and are beneficial to the host, and the purpose of administering probiotics to cancer patients is to reactivate the damaged gut microbiota of the patient, thereby reestablishing the level and function of the failed symbiotic microbiome [90, 91] (Table 3). Ingestion of adequate amounts of these microorganisms significantly improves intestinal crypt survival in mice and other animals by promoting the recovery of healthy gut microbiota and reducing apoptosis, in a protective effect that is partly dependent on TLR-2 and COX-2 [92]. The *Lactobacillus rhamnosus GG* strain (LGG) was the first probiotic studied in the field of oncology. Some previous studies found that LGG can directly regulate the host’s cell proliferation pathways, such as the mTOR or WNT pathways. LGG can also affect the host’s immune system and induce Th1 immune cell polarization through DC recognition, thereby enhancing the antitumor immune response and helping the host to remove newly formed cancer cells early [93–97]. Additionally, when patients were administered a bacterial mixture including two probiotics, *Bifidobacterium longum* (BB536) and *Lactobacillus johnsonii* (La1), these microorganisms were found to adhere to the colonic mucosa, reduce the concentration of intestinal pathogens, and regulate the local formation of an anti-cancer immune environment, as shown by significantly reduced proliferation of CD83-123, CD83-11c, and CD83-HLA-DR subsets in subjects receiving probiotics compared to controls [98].

**Table 3:** Various probiotics in the adjuvant treatment of cancer and their mechanisms of action.

Probiotics	Model	Cancer types	Commentary	References
***A probiotic containing live lactobacillus acidophilus plus bifidobacterium bifidum***	63 patients with cervical cancer received pelvic radiotherapy	Cervical cancer	Reduces the incidence of radiation-induced diarrhea and the need for anti-diarrheal drugs	[179]
***A mixture of Bifidobacterium longum (BB536) and Lactobacillus johnsonii (La1)***	31 patients undergoing elective colorectal cancer resection were enrolled	CRC	The flora adheres to the colonic mucosa, reduces the concentration of pathogens, and regulates the local formation of an anti-cancer immune environment	[98]
***Bifidobacterium lactis and Lactobacillus acidophilus***	15 patients with colon cancer	CRC	Butyrate is produced, which is beneficial to inhibit cell proliferation, reduce IFN-γ-mediated inflammation, promote apoptosis and tumor suppressor gene expression	[180]
***Lactobacillus johnsonii***	Ataxia-telangiectasia mouse model	Lymphoma	Reduces the level of IL-1β and IFN-β, increases the level of TGFβ and IL-10, reduces systemic genotoxicity	[181]
***Lactobacillus helveticus R389 or L89***	BALB/c mouse	Breast cancer	Up-regulates IL-10 and down-regulates IL-6 to inhibit breast tumor cell growth	[182]
***Lactobacillus casei BL23***	C57BL6 mouse	CRC	Adjust caspase-7, caspase-9 and Bik upwards and IL-2 downwards	[183]
***Colon Dophilus (A mixture of 10 different probiotic strains)***	46 patients with colorectal cancer treated with irinotecan-based therapy	CRC	Prevention of diarrhea caused by decreased intestinal glucuronidase activity due to irinotecan	[99]
***Bifilact (Lactobacillus acidophilus LAC-361 and Bifidobacterium longum BB-536)***	246 patients receiving radiotherapy after surgery for pelvic cancer	Pelvic cancer	Reduce radiotherapy-induced pelvic cancer in patients with end-stage grade 2, 3, and 4 diarrhea	[100]
***Four lactic acid bacteria (LAB)***	75 patients undergoing elective colectomy	CRC	Relieves irritable bowel syndrome symptoms, including diarrhea, time to first bowel movements, abdominal pain, and gas	[101]

In terms of mitigating the side effects of anti-cancer treatments, a 2015 clinical trial evaluated the preventive effect of a mixture of 10 different probiotic strains on gastrointestinal toxicity in patients with metastatic colorectal cancer receiving chemotherapy with irinotecan [99]. Microbial agents containing *Lactobacillus acidophilus* and *Bifidobacterium longum* significantly reduced moderate to severe diarrhea during pelvic radiotherapy [100]. The combination of probiotics in patients after CRC surgery may also relieve irritable bowel syndrome [101]. In a 2019 review, 15 studies showed that the combination of *Bifidobacterium longum*, *Lactobacillus acidophilus*, *Bifidobacterium breve*, *Bifidobacterium infantis*, and *Saccharomycetes* reduced the incidence of mucositis in patients who underwent radiation or chemotherapy [102].

### Fecal flora transplantation

FMT from healthy donors has been used to repair dysbacteriosis in the gut and was described in TCM 1700 years ago [103]. In recent years, it has been increasingly used to treat various pathological processes [104–107]. Compared to oral probiotics, it is the most direct and rapid means of manipulating the gut microbiota, and can be administered to patients directly through oral freeze-dried capsules or via gastroscopy or colonoscopy [108–111]. In recent years, evidence has shown that FMT allows breakthroughs in the field of oncology treatment [112–114]. The effectiveness of FMT in reducing local and distant tumorigenesis in the gut was demonstrated in mouse models [115].

Although the clinical application of FMT is still in the experimental stage, it is effective for patients with acute myeloid leukemia and melanoma [116]. In multiple studies on melanoma patients, FMT has been found to alter the gut microbiota, reprogramming the TME by affecting the local immune system and inflammatory response to overcome PD-1-blocker resistance and improve its antitumor efficacy [117–120]. Hematopoietic stem cell transplantation (HSCT), as a relatively mature technology for the treatment of benign and malignant diseases of the blood system, often exposes patients to various complications, including recurrence, infection and graft-versa-host disease (GvHD), whish will lead to a great increase in patient mortality [121–123]. Several studies have shown that FMT is beneficial for GvHD remission in patients who received allogeneic HSCT. In a study of 15 patients who had received HSCT and developed GvHD, restoration of intestinal microbial diversity via FMT addressed steroid-resistant and steroid-dependent GvHD in the gut, with an increased abundance of beneficial bacteria and resolution of diarrhea [124]. Another eight-patient pilot study showed that GvHD patients who received FMT experienced relief of clinical symptoms, including changes in abdominal pain, diarrhea duration, and stool frequency. Gut microbiota composition was also reconstructed, including an increase in *Bacteroidetes*, *Bacteroidaceae*, *Ruminococcaeae*, and *Desulfovibrionaceae* [125].

### Guiding role of gut microbiota in cancer diagnosis and prognosis

In addition to its important role in traditional cancer treatment, gut microbiota are also valuable in the diagnosis and prognosis of cancers such as CRC and LC. In terms of cancer diagnosis, a study in 2017 presented the metagenomic analysis of the CRC fecal microbiome to identify and validate bacterial biomarkers in different ethnic cohorts. This study included patients with CRC and control samples from China, Denmark, France, and Austria and highlighted the potential of fecal metagenomic biomarkers for early CRC diagnosis [126]. Since then, additional studies have found that fecal microbe DNA markers can be used as new tests to screen for colorectal tumors in asymptomatic subjects, either alone or in combination with fecal immunochemical tests. Furthermore, Zhao et al. collected fecal samples from 41 patients with LC and 40 healthy volunteers and analyzed the gut microbiota using 16 S rRNA gene sequencing. They found that *Actinomyces*, *Veillonella*, *Megasphaera*, *Enterococcus*, and *Clostridioides* were more abundant in patients with LC than in healthy individuals. They further demonstrate that gut microbes and their related metabolites as potential biomarkers and therapeutic targets for LC [127]. Gophna et al. examined changes in the gut microbiota and their potential as a biomarker in patients with pancreatic cancer. They compared the microbiomes of pancreatic patients with cancer with precancerous lesions, patients with non-alcoholic fatty liver disease, and healthy controls, and found unique pancreatic cancer-associated gut microbiota signatures. The predominant features were the presence of Clostridiacea, Lachnospiraceae, a lack of Ruminococcaceae, and excessive increases in Veillonellaceae, Akkermansia, and Odoribacter [128]. In conclusion, the gut microbiota profile may become a new effective marker for the early detection of cancer.

In terms of prognosis, many studies have demonstrated that the gut microbiota can be used as a potential prognostic marker of cancer. In a study of a prognostic model of patients with CRC based on age-related genes, Dai et al. found that the risk model was associated with immune status and the gut microbiota in patients with CRC, and that microbiome analysis showed a lower relative abundance of *Bacteroidetes* and *Actinobacteria* in high-risk patients than in low-risk patients. Combined with the results of consensus cluster analysis, *Bacteroides* enrichment in the gut has been associated with a poor prognosis in CRC patients [129]. Colov et al. found that high levels of *Fusobacterium nucleatum* and *Bacteroides fragilis* in the gut are associated with poor postoperative outcomes in patients with CRC [130]. The role of *Fusobacterium nucleatum* as a prognostic marker in patients with CRC has been demonstrated several times. For example, Yamaoka et al. collected 100 CRC tissues and 72 matched normal mucosal tissues and determined that levels of *Fusobacterium nucleatum* could help to predict clinical outcomes in CRC patients, stage IV CRC patients were found to have higher levels of *Fusobacterium nucleatum* [131, 132]. Additionally, Chung et al. demonstrated that specific gut microbiota were related to the prognosis of patients with hepatocellular carcinoma treated with nabuliumab. Specifically, the *Prevotella/Bacteroides* ratio can be used as a prognostic predictor for nivolumab treatment in hepatocellular carcinoma; the higher the ratio, the better the efficacy [133].

### Diet, particularly prebiotics, mediates ecological changes in the gut and their association with cancer

The gut microbiota are an important part of the gut microecology. Diet may be the most powerful regulator of the microbiota in terms of its composition and metabolic function [134]. Previous studies have shown that people with diets rich in complex carbohydrates have significantly increased diversity of gut microbiota. Consumption of wheat-based bread improved body mass index and glucose tolerance, which were associated with *Prevotella* enrichment and increased polysaccharide fermentation capacity. Obese women who ingested a mixture rich in inulin and fructooligosaccharides over a period of time demonstrated enrichment of bacteria producing butyrate that lowered postprandial blood sugar levels. Compared with the high-calorie western diet, the Mediterranean diet is beneficial to the health of the gut microbiota and the host. The latter diet can increase the number of beneficial bacteria, including *Lactobacillus* and *Enterococcus faecalis*, promote an anti-inflammatory environment, reduce oxidative stress, particularly against breast cancer, gastric cancer, and upper gastrointestinal and respiratory cancers [135–142]. Yang et al. demonstrated that a high-fat diet often leads to an increased risk of CRC, which is related to ecological disorder of gut microbiota and intestinal barrier dysfunction, specifically manifested by an increase in *Alistipessp Marseille P5997* and *Alistipessp 5CPEGH6* in the intestine and a decrease in the probiotic *Parabacteroides distasonis* [143]. Compared with fat, dietary fiber derived from fruits, vegetables, and grains can change the density of gut microbiota, such as *Firmicutes*, improve antitumor immunity, and is negatively correlated with the risk of cancer [144].

Prebiotics are food components that are not digested and absorbed by the host and can selectively promote the metabolism and proliferation of probiotics in the gut. Common prebiotics include inulin, fructooligosaccharides, galactose, and some algae [145]. Prebiotics also play an important role in cancer development. A recent case–control study by Turati et al., which included 1,953 patients with histologically confirmed CRC and 4,154 controls, found that CRC risk was negatively associated with dietary intake of galactose [146]. Additionally, as the most common prebiotic, inulin increases the richness of *Bifidobacteria*, *Bacteroides*, and *Akkermansia muciniphila* in the intestinal tract of mice, and is associated with antitumor immunity [147]. In a recent study, Boucher et al. observed that a diet rich in inulin changes the gut microbiota, significantly promotes the growth of *Bifidobacteria*, and based on γδT lymphocyte tumor infiltration processes, also promotes immune control of tumor growth in melanoma, fibrosarcoma, and CRC in a mouse model [148].

### Challenges and prospects

We have summarized the ongoing and completed clinical trials on gut microbiota in cancer treatment (Table 4). In the meantime, our results also raise controversial clinical questions such as how drugs (antibiotics) and environmental factors affect the composition and diversity of the gut microbiota, their interventional role in cancer treatment, and whether monitoring these factors during cancer treatment is necessary. Additionally, when it comes to improving the efficacy of cancer treatment responses by modulating the gut microbiota, it is not clear what composition of gut microbiota are the best for promoting antitumor immune responses. Further treatment options need to be carefully tested in clinical trials. It is also worth noting that most of the mechanisms by which the gut microbiota are involved in regulating cancer have been studied in mice, caution should be exercised when extrapolating these results to humans. Firstly, the intestinal structure and environment of mice and humans are different, as is the diversity of their flora. Secondly, the immune systems of mice and humans react differently to cancer. Thirdly, mice and humans have different diets and environmental exposures. Taken together, several factors contribute to the inability of the current knowledge to predict human clinical responses [149]. Therefore, in future studies, it is necessary to explore the safety, duration, dosage, dosage form, route of administration, combination of drugs, and other aspects of cancer treatment dependent on the gut microbiota more fully, to determine the best treatment plan for cancer.

**Table 4:** Summary of clinical trials exploring the role of gut microbiome regulation in cancer therapy.

Trial Number	Status	Cancer types	Phase	Intervention	Title
NCT03341143	Ongoing	Melanoma	2	FMT with Pembrolizumab	Fecal Microbiota Transplant (FMT) in Melanoma Patients
NCT01790035	Closed	Gastrointestinal Neoplasms	1	Probiotics Lactobacillus rhamnosus GG (LGG)	Probiotic LGG for Prevention of Side Effects in Patients Undergoing Chemoradiation for Gastrointestinal Cancer (LGG)
NCT03885648	Ongoing	Breast cancer	Not Applicable	Observed changes in the microbiome	Breast Cancer and Its Relationship With the Microbiota (MICROMA)
NCT03782428	Closed	Colorectal Cancer	Not Applicable	Probiotic (A mixture of six types of bacteria)	An Evaluation of Probiotic in the Clinical Course of Patients With Colorectal Cancer
NCT03112837	Ongoing	Nasopharynx cancer	Not Applicable	Probiotic (Lactobacillus, Bifidobacterium and Enterococcus)	Effect of Live Combined Bifidobacterium, Lactobacillus and Enterococcus Capsules on Oral Mucositis in Nasopharyngeal Carcinoma Patients Receiving Radiotherapy.
NCT02928523	Closed	Acute myeloid leukemia	2	Autologous Fecal Microbiota Transplantation	Prevention of Dysbiosis Complications With Autologous FMT in AML Patients (ODYSSEE)
NCT03416777	Closed	Colorectal Cancer	Not Applicable	Meat-based diet	Meat-based Versus Pesco-vegetarian Diet and Colorectal Cancer (MeaTIc)
NCT03574051	Ongoing	Thyroid cancer	1	Probiotic (A mixture of three types of bacteria)	the Microbiota Are Associated With Iodine-131 Therapy and Hypothyroidism
NCT02845973	Closed	Colorectal Cancer	Not Applicable	Detected the relative abundance of C. symbiosis in 781 cases by qPCR	Study of Fecal Bacteria in Early Diagnosis of Colorectal Cancer
NCT02944617	Ongoing	Renal Cell Carcinoma	Not Applicable	Dietary supplement: micronutrient-fortified probiotic yogurt	Probiotic Yogurt Supplement in Reducing Diarrhea in Patients With Metastatic Kidney Cancer Being Treated With Vascular Endothelial Growth Factor-Tyrosine Kinase Inhibitor
NCT01538550	Ongoing	Colorectal Cancer	Not Applicable	Metagenomics sequencing analysis of stool samples	Pilot Study of a National Screening Programme for Bowel Cancer in Norway
NCT03316456	Ongoing	Acute Leukemia	Not Applicable	Observational: Stool Sample Collection	Gut Microbiota in Intestinal Barrier Damage in Acute Leukemia Patients Undergoing Inpatient Induction
NCT00549848	Ongoing	Acute Lymphoblastic Leukemia	3	Bacterial 16 S rRNA gene was analyzed by high-depth sequencing	Total Therapy Study XVI for Newly Diagnosed Patients With Acute Lymphoblastic Leukemia
NCT04040712	Ongoing	Renal Cell Cancer	Not Applicable	Donor FMT	Fecal Microbiota Transplantation in Diarrhea Induced by Tyrosine-kinase Inhibitors
NCT03642548	Ongoing	Non-small cell lung cancer	3	Chemotherapy with probiotics	Probiotics Combined With Chemotherapy for Patients With Advanced NSCLC
NCT03353402	Ongoing	Melanoma	1	Donor FMT	Fecal Microbiota Transplantation (FMT) in Metastatic Melanoma Patients Who Failed Immunotherapy
NCT03829111	Ongoing	Renal Cell Carcinoma	1	Chemotherapy with probiotics	CBM588, Nivolumab, and Ipilimumab in Treating Patients With Stage IV or Advanced Kidney Cancer
NCT02843425	Ongoing	Colorectal Cancer	Not Applicable	Diet (dried beans) interferes with intestinal flora	The Beans to Enrich the Gut Microbiome vs. Obesity’s Negative Effects (BE GONE) Trial
NCT03072641	Closed	Colon Cancer	Not Applicable	Probiotic (A mixture of two types of bacteria)	Using Probiotics to Reactivate Tumor Suppressor Genes in Colon Cancer

## Conclusion

Currently, more and more attention has been paid to the research on the composition and function of gut microbiota. Studies on different cancer types and experimental subjects have emphasized that gut microbiota play a dual role in cancer development. In addition, the use of gut microbiota in combination with traditional anti-tumor treatment strategies, as well as the use of probiotics, FMT, and dietary control, can improve the efficacy of anti-cancer treatment, while reducing the occurrence of side effects and improving prognosis. The value of gut microbiota for diagnosis and prognosis of cancer patients is also gradually being confirmed. In conclusion, the artificial control of gut microbiota can promote the development of cancer treatment system in the desired direction, which will provide a scientific basis for the development of more effective anti-cancer treatment programs and the promotion of precision medicine.

## Supplementary information

Supplementary Table

Supplement to the Articl
