# Supplementary material for: Potential effects of gut microbiota on host cancers: focus on immunity, DNA damage, cellular pathways, and anticancer therapy
Source: ISME J. 2023 Aug 8;17(10):1535–51. doi: 10.1038/s41396-023-01483-0 (PMC10504269; doi:10.1038/s41396-023-01483-0)
Supplement: Supplementary file 1 — Supplementary Table [file 41396_2023_1483_MOESM1_ESM.docx]

**Supplementary Table: Experimental evidence for the role of gut microbiota in the occurrence and development of cancer.**

| Experimental model | Methods | Result | References |
| --- | --- | --- | --- |
| Apc(Min/+) mouse model | Flow cytometry | CD11b myeloid cells (mean *3.2 higher cell number, mean *4.0 higher % population) increased in the tumors of Apc(Min/+) mice fed *F. nucleatum* as compared to controls | [1] |
| TE-8 and TE-10 cell lines | Western blotting, IHC | p-p65 is highly expressed in cancer cells treated with *F. nucleatum*, and p65 is localized in the nucleus of ESCC cells treated with *F. nucleatum* | [2] |
| BxPC3, Panc1, HPAC, and Capan1 cell lines | Cell migration assays | *F. nucleatum* infected BxPC3 cells trans-migrated across the membrane at a significantly increased rate compared to uninfected cells（*2） | [3] |
| Colorectal cancer cell line HT29/c1 | qRT-PCR | *Bacteroides fragilis* toxin rapidly induced SMO gene expression in cancer ​cell, resulting in a two- to fourfold increase after a 3- or 6-h exposure | [4] |
| CRC mouse model | Untargeted metabolomics of serum samples | The purine metabolite inosine was the only metabolite that was significantly more abundant (eight to nine fold) in sera from *B. pseudolongum*–monocolonized mice compared with sera from *Colidextribacter* species–monocolonized or GF mice | [5] |
| Squamous cell carcinoma (SCC) VII cells | Enzyme-linked immunosorbent assay, flow cytometry | The levels of IL-4, IFN-γ, IL-10, and Foxp3 were slightly increased in the mice treated with Staphylococcal enterotoxin B; the frequency of IL-9 T cells was significantly higher in mice treated with SEB | [6] |
| HT29 cell lines | Western blotting | Wild-type EPEC causes a dramatic depletion of cell DNA mismatch repair proteins in HT29 cells after 4 to 5 h | [7] |
| Mouse intestinal loop model | Immunohistology, western blotting | Significant numbers of γH2AX foci were found in the nuclei of enterocytes exposed to *E. coli*. γH2AX foci were found in 22.7% of enterocytes infected with wild-type *E. coli*, three times more than in control group | [8] |
| C57BL/6 mouse model | Immunostaining of gastric tissues | Immunostaining of gastric tissues indicated increased SMO protein levels in epithelial cells from patients infected with *H. pylori*. Both cytoplasmic and nuclear localization of SMO was detected on high power images. There was also strong staining for the DNA damage marker, 8-OHdG;in gastric biopsies from patients infected with H. pylori | [9] |
| Human gastric adenocarcinoma cell line AGS | Western blotting | Treatment of cells with 1μg/ mL DNA damage (Dox) 5 h after *H. pylori* infection for 1.5 h caused a dramatic increase in p53 levels and up-regulated downstream effectors p21 and Bax | [10] |
| Wistar rat model | Ferricytochrome c reduction assay | *E. faecalis* produces 17.2 ± 0.3 nmol of extracellular superoxide per min per 109 colony-forming units (CFU) as measured by the ferricytochrome C reduction assay | [11] |
| BALB/c GF mouse model | LC-MS | Mutant and wild-type *B. fibrisolvens* strains were cultured in medium supplemented with oligosaccharide/inulin, and butyrate levels in mutant medium were observed to be 7 times lower than those in wild-type cultures | [12] |
| Human CRC cell line LS174T and murine colon cancer MC38 cells | Cell Counting, RNA extraction and quantitative Real-time PCR and cell cycle analysis | *P. gingivalis* can significantly promote S1 and MC38 cell proliferation (P < 0.05) and increase the percentage of CRC cells in S phase; Exogenous Gingipains were added at the beginning of infection, the cell vitality of KDP136 group with exogenous Gingipains (5 U/L) is significantly higher than control group; KRAS, BRAF, MEK2, ERK2, C-fos and AP1 levels in the *P. gingivalis* groups were higher than those observed in the control group after exposure to bacteria for different amounts of time | [13] |
| Mouse colon cancer cell line CT26、CRC mouse model | Kinetic quantification of cytokines and chemokines | The outer membrane vesicles (OMV) of 5 μg E. coli W3110 eliminated the tumor completely at 15 days. Measure the kinetic quantification of cytokines in blood serum and tumor tissue until 48 h after the intravenous injection of OMVs, IFN-γ and CXCL10 increased time dependently in the tumor tissue | [14] |
| Caco2, SKCO-1 and SW620 cells, | SRB assay | The numbers of colon cancer cells were significantly lower in the conditioned media from the Lactobacillus GG, L. casei, L. coryniformis and L. fermentis treated groups than in the control group. | [15] |
| Human oral keratinocytes and HSC-3 human oral squamous cell carcinoma cell line | Western blot analysis, flow cytometric assay | The flow cytometry experiment revealed that the apoptotic cells (sub-G1 DNA content) in the control, geniposide-L, LGG-geniposide-L, geniposide-H and LGG-geniposide-H-treated HSC-3 cells were 2.2±0.3, 9.7±0.4, 14.3±0.6, 24.8±1.8 and 32.6±2.3%, respectively; The high concentration of geniposide and LGG resulted in the highest Fas, IκB-α, p53 and p21 expression. | [16] |
| HepG2 cell line | Gene expression analysis | The Bax gene expression in groups exposing to 50 and 100 μg/ml LDEVs was 2 and 1.58 times more than the negative group. The apoptotic index was significantly increased after treating with 50 and 100 μg/ml LDEVs. Nearly, 2.05 and 2.28 times increase was seen at the concentrations of 50 and 100 μg/ml LDEVs in comparison with the control group. | [17] |
| SPF C57BL/6 and BALB/c mice | 16S rRNA sequencing | Increased CXCL16 mRNA levels in germ-free mouse livers (about twice) compared to *Clostridium scindens* colonization | [18] |
| Pax5+/– mice, and Sca1-ETV6-RUNX1 littermates | Statistical analysis | In a total of 23 mice with pB-ALL defects, 11 developed pB-ALL between 11 and 20 months of age, while none developed in germ-free mice (n = 12) | [19] |
| Melanoma mouse model with subcutaneous injection of B16.SIY cell line | ELISPOT and flow cytometry | Transplantation of *Bifidobacteria* can induce the activation of peripheral tumor-specific T cells and increase the accumulation of antigen-specific CD8+T cells in the tumor | [20] |
| HT29/c1 and T84 cell lines | qRT-PCR | BFT rapidly induced SMO gene expression in both cell types, resulting in a two- to fourfold increase after a 3- or 6-h exposure | [21] |

**References**

1. Kostic, A.D., et al., *Fusobacterium nucleatum potentiates intestinal tumorigenesis and modulates the tumor-immune microenvironment.* Cell Host Microbe, 2013. **14**(2): p. 207-15.

2. Nomoto, D., et al., *Fusobacterium nucleatum promotes esophageal squamous cell carcinoma progression via the NOD1/RIPK2/NF-κB pathway.* Cancer Lett, 2022. **530**: p. 59-67.

3. Udayasuryan, B., et al., *Fusobacterium nucleatum induces proliferation and migration in pancreatic cancer cells through host autocrine and paracrine signaling.* Sci Signal, 2022. **15**(756): p. eabn4948.

4. Wu, S., et al., *Bacteroides fragilis enterotoxin induces intestinal epithelial cell secretion of interleukin-8 through mitogen-activated protein kinases and a tyrosine kinase-regulated nuclear factor-kappaB pathway.* Infect Immun, 2004. **72**(10): p. 5832-9.

5. Mager, L.F., et al., *Microbiome-derived inosine modulates response to checkpoint inhibitor immunotherapy.* Science, 2020. **369**(6510): p. 1481-1489.

6. Miao, B.P., et al., *Inhibition of squamous cancer growth in a mouse model by Staphylococcal enterotoxin B-triggered Th9 cell expansion.* Cell Mol Immunol, 2017. **14**(4): p. 371-379.

7. Maddocks, O.D., K.M. Scanlon, and M.S. Donnenberg, *An Escherichia coli effector protein promotes host mutation via depletion of DNA mismatch repair proteins.* mBio, 2013. **4**(3): p. e00152-13.

8. Cuevas-Ramos, G., et al., *Escherichia coli induces DNA damage in vivo and triggers genomic instability in mammalian cells.* Proc Natl Acad Sci U S A, 2010. **107**(25): p. 11537-42.

9. Chaturvedi, R., et al., *Spermine oxidase mediates the gastric cancer risk associated with Helicobacter pylori CagA.* Gastroenterology, 2011. **141**(5): p. 1696-708.e1-2.

10. Buti, L., et al., *Helicobacter pylori cytotoxin-associated gene A (CagA) subverts the apoptosis-stimulating protein of p53 (ASPP2) tumor suppressor pathway of the host.* Proc Natl Acad Sci U S A, 2011. **108**(22): p. 9238-43.

11. Huycke, M.M., V. Abrams, and D.R. Moore, *Enterococcus faecalis produces extracellular superoxide and hydrogen peroxide that damages colonic epithelial cell DNA.* Carcinogenesis, 2002. **23**(3): p. 529-36.

12. Donohoe, D.R., et al., *A gnotobiotic mouse model demonstrates that dietary fiber protects against colorectal tumorigenesis in a microbiota- and butyrate-dependent manner.* Cancer Discov, 2014. **4**(12): p. 1387-97.

13. Mu, W., et al., *Intracellular Porphyromonas gingivalis Promotes the Proliferation of Colorectal Cancer Cells via the MAPK/ERK Signaling Pathway.* Front Cell Infect Microbiol, 2020. **10**: p. 584798.

14. Kim, O.Y., et al., *Bacterial outer membrane vesicles suppress tumor by interferon-γ-mediated antitumor response.* Nat Commun, 2017. **8**(1): p. 626.

15. Konishi, H., et al., *Probiotic-derived ferrichrome inhibits colon cancer progression via JNK-mediated apoptosis.* Nat Commun, 2016. **7**: p. 12365.

16. Cheng, Z., et al., *Lactobacillus raises in vitro anticancer effect of geniposide in HSC-3 human oral squamous cell carcinoma cells.* Exp Ther Med, 2017. **14**(5): p. 4586-4594.

17. Behzadi, E., H. Mahmoodzadeh Hosseini, and A.A. Imani Fooladi, *The inhibitory impacts of Lactobacillus rhamnosus GG-derived extracellular vesicles on the growth of hepatic cancer cells.* Microb Pathog, 2017. **110**: p. 1-6.

18. Ma, C., et al., *Gut microbiome-mediated bile acid metabolism regulates liver cancer via NKT cells.* Science, 2018. **360**(6391).

19. Vicente-Dueñas, C., et al., *An intact gut microbiome protects genetically predisposed mice against leukemia.* Blood, 2020. **136**(18): p. 2003-2017.

20. Sivan, A., et al., *Commensal Bifidobacterium promotes antitumor immunity and facilitates anti-PD-L1 efficacy.* Science, 2015. **350**(6264): p. 1084-9.

21. Goodwin, A.C., et al., *Polyamine catabolism contributes to enterotoxigenic Bacteroides fragilis-induced colon tumorigenesis.* Proc Natl Acad Sci U S A, 2011. **108**(37): p. 15354-9.
