# Supplementary material for: Potential effects of gut microbiota on host cancers: focus on immunity, DNA damage, cellular pathways, and anticancer therapy
Source: ISME J. 2023 Aug 8;17(10):1535–51. doi: 10.1038/s41396-023-01483-0 (PMC10504269; doi:10.1038/s41396-023-01483-0)
Supplement: Supplementary file 2 — Supplement to the Articl [file 41396_2023_1483_MOESM2_ESM.pdf]

Supplement to the article.

1. Gut microbiota are associated with chromosomes stability, epigenome change, and microRNA

1.1 Gut microbiota and chromosomal stability

To assess the relationship between gut microbiota and chromosome stability, the researchers exposed intestinal epithelial cells to macrophages co-cultured with *Enterococcus faecalis* and found the percentage of aneuploidy cells increased significantly compared with the control group. Subsequent intraperitoneal injection of these epithelial cells into the mice resulted in the formation of distinct lumps at the injection site [1]. Subsequent studies have also demonstrated that *E. faecalis* produces extracellular superoxide and reactive oxygen species through the autoxidation of cell membrane-associated demethylmethquidone, which may be an important cause of Chromosomal Instability associated with colorectal cancer. The results of the comet test showed that colonic cells colonized by peroxide-producing *E. faecalis* showed significantly increased DNA damage compared with control rats (Figure 2) [2].

1.2 Gut microbiota and epigenome change

In addition to the genome, epigenome changes, including high and low methylation levels and the dynamic balance of acetylation and deacetylation are related to the occurrence of cancer [3-5]. Gut microbiota also play an important role in this process. For example, compared to conventional mice, germ-free mice show high methylation of the proto-oncogene *Bcl3* and reduced gene expression, while the expression of tumor suppressor gene Rb1 was increased [6]. By administering antibiotics to premature piglets, bacterial colonization can affect the nascent immature gut through DNA methylation, which is essential for gut health [7]. Treatment of mature or immature intestinal epithelial cells with probiotic species (*Lactobacillus acidophilus* and *Bifidobacteria*) or *Klebsiella* species results in methylation alterations in hundreds of relevant genes [8]. In terms of acetylation of

histones, the *Escherichia coli* strain contains a genomic island called "PKS", which encodes the production of a polyketide genotoxin that causes phosphorylated histone H2AX foci in mouse intestinal epithelial cells, which may lead to the development of sporadic CRC [9]. Inoculation of germ-free mice with butyrate-secreting *Butyrivibrio fibrisolvens* largely prevents tumor formation, with butyrate acting as a histone deacetylase inhibitor, increasing the degree of acetylation of intracellular histones and inhibiting the proliferation of tumor cells. Quantitative ChIP assays and RT-qPCR were performed to assess pan-histone 3 acetylation (H3ac) levels and found that the levels of H3ac were significantly higher in the tumors of butyrate-treated mice than in the control group [10]. SCFAs, as bacterial derivatives, also have epigenetic anticancer effects. For instance, butyric acid and propionic acid secreted by *Propionibacterium* can inhibit histone deacetylases of host tumor cells, and have antitumor effects against CRC and lymphoma in vitro and in vivo (Figure 2) [11, 12].

1.3 Gut microbiota and microRNA

Additionally, some studies have shown that the gut microbiota also influences the expression of non-coding RNAs such as microRNAs (miRNAs). For example, levels of miR-21-5p expression are higher in the intestinal tract of regular mice than in those of germ-free mice. MiR-21-5p regulates tumor growth, including esophageal cancer and lung adenocarcinoma, in the host through various pathways. The co-culture of HT-29 and SW480 cells (two CRC cell lines) with *Bacteroides acidifaciens* and *Lactobacillus johnsonii* also resulted in the upregulation of miR-21-5p in the cells. Based on the results of qRT-PCR, miR-21-5p expression was higher (twice or even more) in small intestinal epithelial cells of microbiota colonized mice compared to GF mice [13, 14].

2. Tumor-related signaling pathways

2.1 Oncogenic tumor-related signals of gut microbiota

The gut microbiota participating in the regulation of tumor-related signaling pathways are mostly cancer promoting. CagA, a protein produced by *Helicobacter pylori*, its presence induces the degradation of p53 in gastric epithelial cells, leading to the occurrence of gastric cancer. Additionally,

CagA can bind to many cellular enzymes, including the tyrosine phosphatase SHP-2 [15]. E-cadherin is a tumor suppressor present in cell membranes, acting through β-catenin, whose EC5 domain of an 11-amino acid (aa) suppressor peptide can inhibit pathogen invasion and eliminate a range of host reactions triggered thereby, including tumor growth and inflammation. FadA, an adhesive protein secreted by *Clostridium*, is the main protein that mediates its attachment and invasion. FadA binds to an 11-aa region on E-cadherin, inhibits its activity, and drives the occurrence of CRC and other cancers [16-18]. CagA can also interact with E-cadherin secreted by host epithelial cells, disrupt intercellular connections, activate catenin signaling, and increase the risk of host cell carcinogenesis [19]. Similarly, MP secreted by *Bacteroides fragilis* and toxins released by *Salmonella* and *Fusoplasma spp.* have a similar effect, amplifying tumor growth by activating the E-cadherin/WNT/β-Catenin pathway [16, 20, 21]. Additionally, a recent study has found that BFT induces the expression and basal secretion of CXC chemokines, such as CXCL8 and CXCL1, which in turn stimulates the expression and production of IL-8 and GRO-α. In this process, activation of NF-kB signaling by BFT, by inducing p65 and p50 heterodimers may be a key regulatory step [22]. Due to the increase in TLR expression in tumor cells and the change in the stratification of tumor-associated gut microbiota, calcineurin and NFAT transcription factors, of which are involved in inflammatory pathways, are expressed in intestinal epithelial cells and are selectively activated in tumor tissues. After activation by microbial-derived TLR ligands, calcineurin allows cancer stem cells to survive, proliferate, and promote tumor development in an NFAT-dependent manner [23]. *Pseudomonas gingivalis* is the primary pathogen causing periodontitis. Clinical studies have found a large number of *P. gingivalis* in the fecal samples and colorectal mucosa of patients with CRC. The virulence factors of *P. gingivalis* include fimbria, hemagglutinin, capsules, lipopolysaccharides, and gingival proteases. Among them, gingival proteases play an important pathogenic role in the body and are a unique cysteine endopeptidase family that is widely expressed on the outer membrane of *Pseudomonas gingivalis* or is secreted into the extracellular environment by activating the

MAPK/ERK signaling pathway to invade host cells and promote CRC cell proliferation (Figure 3) [24, 25].

2.2 Anticancer tumor-related signals of gut microbiota

The gut microbiota can exert a cancer-suppressing effect by influencing tumor-related signaling pathways. GPR109A is a G-protein-coupled receptor that exists on the lumen surface of colon epithelial cells, recognizes bacterial metabolites such as nicotinate and butyrate, and further mediates the release of GPR109A-dependent IL-18 in the colon epithelium, inhibiting the occurrence of colorectal cancer [26]. The gut microbiota produce extracellular vesicles (outer membrane vesicles, OMVs) and transports cytokines, metabolites, and antigen, which can trigger inflammatory responses in homeostasis and pathological processes of the body, provide proliferative signals, enhance immune effects, and trigger profound phenotypic changes in the tumor microenvironment (TME). OMVs extracted from *Lactobacillus rhamnosus GG,* has a direct antitumor effect on the growth of liver cancer cells in this manner [27-30]. As a gram-negative bacterium, *Bacteroides multiforme* secretes OMVs that cross the intestinal epithelial barrier and promotes host–flora interaction, re-editing of the TME, and differentiation of Th0 cells towards Th1 to secrete CXCL10 and IFN-γ, which plays a role in maintaining intestinal barrier homeostasis and suppressing cancer [31]. In mouse models, *Lactobacillus casei*-derived iron chromate is directly activated through the JNK pathway to trigger apoptosis of tumor cells, SRB experiments showed that iron chromate (concentrations >100 ng ml^{-1}) reduced cell proliferation in colon cancer cells (Caco2 and SW620) (Figure 3) [32].

2.3 Gut microbiota metabolites and tumor-related signals

Both the constituent components of the gut microbiota and secreted products involved in the metabolism of the body are involved in the development and progression of tumors [33, 34]. They may take part in the regulation of cellular pathways, affect enzyme activity, and play a dual role in promoting or suppressing cancer. Here, we summarize their association with tumor-related signaling

pathways and describe them in detail.

LPS is the most important glycolipid outer-membrane component of gram-negative bacteria, which effectively activates immune cells by binding to TLR-4 on the cell surface, activating transcription factors (NF-kB) and protein kinases (p38 kinase), causing effects such as cancer invasion or angiogenesis. LPS activates NF-kB through TLR-4, upregulates urokinase plasminogen activator (u-PA) in a dose-dependent manner and enhances vitreous protein adhesion and tumor extracellular matrix invasion in tumor cells. Moreover, these effects were improved by inhibiting u-PA and u-PAR [35]. In mouse tumor models, LPS-induced inflammation promotes angiogenesis by up-regulating vascular endothelial growth factor (VEGF), thereby affecting the invasion and migration of tumor cells [36].

SCFAs, particularly butyrate, exhibit different effects on normal and cancer cells. Butyrate is considered to be an inducer of MDR1 expression, which is involved in drug-resistance in thyroid cancer and affects the antitumor treatment effect [37]. Butyrate also affects epigenetic modification by blocking histone deacetylation. At appropriate concentrations, butyrate can increase the degree of cell differentiation in colorectal cancer and reduce the proliferation of cancer cells, the researchers reported [38]. Additionally, *Bifidobacteriaceae*, *Vibrio desulfurae*, and *Bacteroidetes* promote the production of polyunsaturated fatty acids (PUFAs) from dietary fats. The antiproliferative effect of PUFAs is attributed to the generation of oxidative environment, the increase of apoptosis, and the disruption of cancer cell proliferation cycle to varying degrees, which is contrary to the findings of DNA damage caused by the gut microbiota mentioned above [39-41]. For example, conjugated linoleic acid (CLAs), a product of biological hydrogenation of linoleic acid by bacteria expressing large amounts of linoleic isomerase, has significant anti-proliferative effects by decreasing the expression of MMP-2, MMP-9, VEGF, and inflammatory mediators such as TNF-α, IL-1β, and C-reactive protein. In a mouse model of epidermal carcinoma, the incidence of tumor in mice receiving CLAs locally was lower than that in control mice, and the tumor load in CRC rats was

reduced by gavage of CLAs (Figure 3) [33, 41, 42].

3. Moderating effect of gut microbiota on radiotherapy and chemotherapy

3.1 Radiotherapy

Radiotherapy is a method of treating tumors with radiation that induces genotoxicity in tumor cells, and has been shown to have long-lasting effects on localized tumors [43, 44]. A 2004 study revealed that radiation therapy can affect the gut microbiota. Experiments at that time demonstrated the effect of local ionizing radiation on the diversity of the microbiota in the ileal segment of mice, and showed that radiation caused a decrease in the number of *Enterobacteriaceae*. After 24 h, the effects of ionizing radiation disappeared and the flora species recovered [45]. Recent studies have evaluated long-term changes in the gut microbiota after radiation therapy. After 10 months of irradiation, the abundance of *Bacteroidetes* in the gut microbiota of irradiated mice increased and the abundance of *Clostridium difficile* decreased compared to that in control mice [46].

In addition to the effects of radiotherapy on the gut microbiota, the gut microbiota also affect the efficacy and side effects of radiotherapy. In patients with melanoma undergoing radiotherapy, the efficacy of the treatment depends on the composition of the gut microbiota, translocation, and the TLR4 signaling pathway of the host [47, 48]. A 2020 study reported that *Clostridium*-derived immunosuppressive metabolites, including butyric acid and propionic acid, resisted radiation therapy in tumor lesions, while vancomycin reversed this phenomenon by increasing antigen presentation by DCs and $CD8^+$ T-cell activation [47]. In terms of toxic side effects, radiation therapy can cause significant damage to bone marrow and epithelial cells, leading to complications such as diarrhea, colitis, and bone diseases [49, 50]. Gastrointestinal complications caused by acute radiation, such as diarrhea and mucositis, are major treatment barriers to radiotherapy, with an incidence of 80% in pelvic radiotherapy and > 90% in head and neck cancer [51]. Most of these toxic side effects are caused by direct exposure to ionizing radiation, ROS production, and localized inflammatory outbreaks [52]. The probiotic *Lactobacillus rhamnosus* in the gut relocates stem cells to the basilar

part, thereby protecting the intestine from radiation-induced cell damage [53]. *Acidophilus*, *Bifidobacteria*, and *Streptococcus spp.* have been shown to relieve gastrointestinal side effects, such as diarrhea, abdominal pain, and nausea in clinical trials that inhibit the cytotoxic effects of radiotherapy [54-57]. The importance of the gut microbiota in intestinal endothelial radiation sensitivity was revealed in a study on a mouse model of hematopoietic stem cell transplantation with total body irradiation as an adjunct treatment regimen, which found that radiation-lethal enteritis occurred less frequently in the small intestine of germ-free mice [58].

3.2 Chemotherapy

Chemotherapy is a treatment that uses chemotherapeutic drugs to kill cancer cells. Members of the gut microbiota of mice produce TLR agonists, which promote an increase in the oxidative stress environment and the death of tumor cells. When the gut microbiota are dysbiotic, less ROS are produced, and the response of mice to chemotherapy also decreases [59]. For example, glutathione peroxidase (GPX-1 and GPX-2) is the main enzyme present in intestinal epithelial cells, which promotes the decomposition of H_2O_2 and reduces the oxidative environment, thereby protecting the structure and function of the cell membrane from peroxide interference and damage. Experiments have proven that knock-out of *Gpx1* and *Gpx2* in mice make the animals highly sensitive to inflammation and chemotherapy, while the presence of *Helicobacter pylori* and other gut microbiota significantly inhibit the function of GPX-1 and GPX-2, thus improving the efficacy of chemotherapy [60].Although chemotherapy is still the primary treatment for most cancer patients, individual differences in patients' sensitivity to chemotherapy drugs have always posed clinical challenges. Many studies have focused on how to regulate the relationship between the efficacy of chemotherapy drugs and their toxic side effects. A previous study reported that the gut microbiota and a variety of chemotherapy drugs, including irinotecan, cyclophosphamide, fluorouracil, and platinum-based anticancer drugs, interact through a series of chemical signal cascades [61]. For example, in the process of exploring how chemotherapeutic drugs modulate the gut microbiota, it was found that

mice treated with irinotecan (CPT11) had significantly lower gut microbiota abundance and significantly higher levels of *Proteus spp.*, *Porphyromonas spp.*, and *Clostridium difficile* than control mice. Mechanistically, CPT11 can trigger innate immune responses, accelerate mucin excretion in goblet cells and affects gut microbiota by reducing the number of adhesion sites [62-64].

In recent years, different studies have attempted to explore how the gut microbiota affect the efficacy of chemotherapy. In a clinical trial of epirubicin treatment after transurethral bladder cancer resection, researchers found that daily administration of *Lactobacillus casei* along with epirubicin treatment significantly improved recurrence-free survival as compared with epirubicin alone [65]. Cisplatin in combination with lactic acid bacteria improves the response of cancer mouse models to anticancer therapy by activating pro-apoptotic genes and enhancing host immune responses, including low expression of VEGFA, high expression of BAX and CDKN1B, and elevated serum levels of IL-6 and IFN-γ [66, 67]. Widely used chemotherapy drugs, such as cyclophosphamide, combined with *Lactobacillus johnsonii*, lead to the transformation of naïve T-cells to Th17 cells, producing the $CD8^+$ T-cell effect of INF and improving chemotherapy efficacy in tumor-bearing mice [68]. In LC model mice treated with cisplatin combined with vancomycin, ampicillin, and other antibiotics, the tumor volume growth rate was significantly accelerated. In contrast, tumor tissue in mice treated with cisplatin and *Lactobacillus* grew more slowly, it's related to the gut microbiota regulating the expression of genes such as VEGFA, BAX, and CDKN1B in tumors and enhancing adaptive immune responses [66]. Similarly, *Proteobacteria* are involved in regulating the effectiveness of gemcitabine in human pancreatic ductal adenocarcinoma tumors [69].

Regarding the toxic side effects of chemotherapy drugs, oral administration of *Bifidobacterium* can significantly reduce intestinal damage during 5-fluorouracil (5-FU) administration in mice, such as shortening of intestinal villi, diarrhea, release of inflammatory factors, and improvement of weight loss in mice. Similar results were found in mouse CRC models treated with irinotecan in combination with probiotics such as *Lactobacillus rhamnosus*, *Lactobacillus acidophilus*,

Lactobacillus casei, *Lactobacillus plantarum*, and *Streptococcus thermophiles* [70-72]. In an in vitro model of CRC, *Lactobacillus plantarum* increased the cytotoxicity of 5-FU through apoptosis and reduced the number of stem-like cancer cells [73]. Certain gut microbiota, such as *Proteobacteria,* may stimulate resistance to commonly used cancer drugs, such as irinotecan, oxaliplatin, cyclophosphamide, gemcitabine, and anthracyclines, and can also attenuate gemcitabine's anticancer activity by producing a bacterial enzyme isoform of cytidine deaminase, which is reversed by the antibiotic ciprofloxacin [74]. By activating TLRs and stimulating miRNA expression, *Fusobacterium nucleatum* also induces resistance to oxaliplatin [75].

We hypothesize that the analysis of gut microbiota before the start of chemotherapy can predict the efficacy of individual chemotherapy drugs and provide a basis for the selection of treatment. For example, a prospective study conducted in 2021 examined the predictive value of gut microbiota for chemotherapy and radiotherapy in patients with rectal cancer [76].

3.3 Immunological therapy

An important mechanism for immune escape of tumor cells is through overexpression of immune checkpoint receptors such as CTLA-4 and programmed death ligand 1, PD-1. Multiple studies have shown that the gut microbiota can stimulate the antitumor effects of CTLA-4 blockers and PD-1 blockers [77-80]. In 2007, studies using mouse models showed that the gut microbiota could stimulate an antitumor immune response. In 2015, the gut microbiota were first linked to ICI responses in mice, and subsequent studies have shown that the diversity of the gut microbiota can predict the efficacy of ICI immunotherapy. Several prospective and retrospective studies from 2019 to 2021 suggested that the gut microbiota significantly affect the prognosis and drug resistance of patients with cancer treated with ICIs [48, 81-90]. In a recent study on pituitary adenoma, Nie et al. found differences in the immune characteristics and gut microbiota (*Bacteroidetes*, *Blautia*, *Enterococcus*, and *Metamonada*) diversity among patients with growth hormone-secreting pituitary adenoma (GHPA), non-functional pituitary adenoma (NFPA), and healthy controls. To analyze the

correlation between gut microbiota and immunity in pituitary adenoma further, Nie et al. administered fecal supernatants from patients in the above three groups into the stomachs of mice (10 mice per group). They found that PD-L1-expressing cells in GHPA-FMT mice were more diffuse than those in the other two groups: the proportion of PD-L1-positive tumors in GHPA and NFPA samples was 64% and 4%, respectively, and the tumor growth was more rapid in GHPA-FMT mice [91]. This evidence indicates that the gut microbiota have a significant impact on the antitumor immunity of the body, and even on the effect of cancer immunotherapy.

Antibiotics have been reported to reduce the efficacy of immune checkpoint blockade in multiple cancer types, such as melanoma, non-small cell LC, urothelial carcinoma, and other solid organ tumors, by eliminating gut microbiota diversity [92-97]. Most floras that responded to ICIs belonged to the phyla *Firmicutes*, *Bacteroidetes*, *Actinomycetes*, *Proteobacteria*, and *Verrucomicrobia* [98]. For example, the presence of *Bacteroides fragilis* and *Burkholderia cepacia* significantly enhanced the effectiveness of CTLA4 blockers in inhibiting sarcoma tumor growth in mice [80]. The efficacy of anti-PD-L1 in the treatment of melanoma in mice was also improved in the presence of *Bifidobacteria* [81], *Bacteroides fragilis*, and *Burkholderia cepacia* [82]. Patients with high *Clostridium spp.*, *Ruminococcus*, or *Enterococcus faecalis* abundances have higher levels of $CD4^+$ and $CD8^+$ T-cells and therefore respond better to immunotherapy [99], In contrast, a good antitumor immune response is associated with the enrichment of tumor-infiltrating lymphocytes and a decrease in MDSCs, while enrichment of *Bacteroidetes spp.* in the gut often leads to an increase in Treg cells and MDSCs, and such patients respond poorly to ICIs [100].

Because of subsequent immune dysregulation, the use of ICIs can cause strong side effects in patients, such as intestinal inflammation; therefore, the duration of this therapy will be limited in a large number of patients. In animals, gavage with *Bacteroides fragilis* and *Burkornella cepacia* can reduce the toxic side effects associated with immunotherapy [80, 101]. The presence of *Bacteroidetes* in the intestine reduces the incidence of colitis induced by CTLA-4 treatment in melanoma patients,

whereas the presence of *Fusobacterium* and other Firmicutes more frequently causes an increased incidence of colitis associated with antitumor therapy in patients with metastatic melanoma [102, 103].

CD47 is expressed on tumor cells and sends a "no phagocytosis" signal to macrophages, mediating immune escape in tumor cells; thus, anti-CD47 blockers have increasingly received attention, but the results of such investigations have not been satisfactory. *Bifidobacteria* accumulate in CRC tumors and stimulate type I interferon signaling in a STING-dependent manner (STING is an interferon-stimulating factor) to improve the functional activation of DCs, thereby promoting CD47-dependent immune responses [104-106].

Mucin domain protein-3 (TIM-3) blockade is another anticancer immunotherapy that aims to alleviate T-cell exhaustion and inhibit Treg cells, thereby enhancing antitumor immunity. Recent studies have shown that antibiotic induced microbiota disorders can reduce the effectiveness of TIM-3 blocking therapy, while it can be restored by altering the composition of the gut microbiota with FMT and the application of probiotics such as *Enterococcus*, or *Lactobacillus* [97].

References

1. Wang X., Yang Y. and Huycke M.M., *Commensal bacteria drive endogenous transformation and tumour stem cell marker expression through a bystander effect.* Gut, 2015. **64**(3): p. 459-68.
2. Huycke M.M., Abrams V. and Moore D.R., *Enterococcus faecalis produces extracellular superoxide and hydrogen peroxide that damages colonic epithelial cell DNA.* Carcinogenesis, 2002. **23**(3): p. 529-36.
3. Timp W. and Feinberg A.P., *Cancer as a dysregulated epigenome allowing cellular growth advantage at the expense of the host.* Nat Rev Cancer, 2013. **13**(7): p. 497-510.
4. Baylin S.B. and Jones P.A., *A decade of exploring the cancer epigenome - biological and translational implications.* Nat Rev Cancer, 2011. **11**(10): p. 726-34.
5. Weisenberger D.J., Lakshminarasimhan R. and Liang G., *The Role of DNA Methylation and DNA Methyltransferases in Cancer.* Adv Exp Med Biol, 2022. **1389**: p. 317-348.
6. Pan W.H., Sommer F., Falk-Paulsen M., Ulas T., Best P., Fazio A., et al., *Exposure to the gut microbiota drives distinct methylome and transcriptome changes in intestinal epithelial cells during postnatal development.* Genome Med, 2018. **10**(1): p. 27.
7. Pan X., Gong D., Nguyen D.N., Zhang X., Hu Q., Lu H., et al., *Early microbial colonization affects DNA methylation of genes related to intestinal immunity and metabolism in preterm pigs.* DNA Res, 2018. **25**(3): p. 287-96.
8. Cortese R., Lu L., Yu Y., Ruden D. and Claud E.C., *Epigenome-Microbiome crosstalk: A potential new paradigm influencing neonatal susceptibility to disease.* Epigenetics, 2016. **11**(3): p. 205-15.
9. Cuevas-Ramos G., Petit C.R., Marcq I., Boury M., Oswald E. and Nougayrède J.P., *Escherichia coli induces DNA damage in vivo and triggers genomic instability in mammalian cells.* Proc Natl Acad Sci U S A, 2010. **107**(25): p. 11537-42.
10. Donohoe D.R., Holley D., Collins L.B., Montgomery S.A., Whitmore A.C., Hillhouse A., et al., *A gnotobiotic mouse model demonstrates that dietary fiber protects against colorectal tumorigenesis in a microbiota- and butyrate-dependent manner.* Cancer Discov, 2014. **4**(12): p. 1387-97.
11. Wei W., Sun W., Yu S., Yang Y. and Ai L., *Butyrate production from high-fiber diet protects against lymphoma tumor.* Leuk Lymphoma, 2016. **57**(10): p. 2401-8.
12. Ramos Meyers G., Samouda H. and Bohn T., *Short Chain Fatty Acid Metabolism in Relation to Gut Microbiota and Genetic Variability.* Nutrients, 2022. **14**(24).
13. Nakata K., Sugi Y., Narabayashi H., Kobayakawa T., Nakanishi Y., Tsuda M., et al., *Commensal microbiota-induced microRNA modulates intestinal epithelial permeability through the small GTPase ARF4.* J Biol Chem, 2017. **292**(37): p. 15426-15433.
14. Wang G., Zhou Y., Chen W., Yang Y., Ye J., Ou H., et al., *miR-21-5p promotes lung adenocarcinoma cell proliferation, migration and invasion via targeting WWC2.* Cancer Biomark, 2020. **28**(4): p. 549-559.
15. Asmamaw M.D., Shi X.J., Zhang L.R. and Liu H.M., *A comprehensive review of SHP2 and its role in cancer.* Cell Oncol (Dordr), 2022. **45**(5): p. 729-753.
16. Rubinstein M.R., Wang X., Liu W., Hao Y., Cai G. and Han Y.W., *Fusobacterium nucleatum promotes colorectal carcinogenesis by modulating E-cadherin/β-catenin signaling via its FadA adhesin.* Cell Host Microbe, 2013. **14**(2): p. 195-206.
17. Ramirez Moreno M., Stempor P.A. and Bulgakova N.A., *Interactions and Feedbacks in E-Cadherin*

Transcriptional Regulation. Front Cell Dev Biol, 2021. **9**: p. 701175.

18. Castagnoli L., Tagliabue E. and Pupa S.M., *Inhibition of the Wnt Signalling Pathway: An Avenue to Control Breast Cancer Aggressiveness.* Int J Mol Sci, 2020. **21**(23).
19. Liatsos C., Papaefthymiou A., Kyriakos N., Galanopoulos M., Doulberis M., Giakoumis M., et al., *Helicobacter pylori, gastric microbiota and gastric cancer relationship: Unrolling the tangle.* World J Gastrointest Oncol, 2022. **14**(5): p. 959-972.
20. Silva-García O., Valdez-Alarcón J.J. and Baizabal-Aguirre V.M., *Wnt/β-Catenin Signaling as a Molecular Target by Pathogenic Bacteria.* Front Immunol, 2019. **10**: p. 2135.
21. Lu R., Wu S., Zhang Y.G., Xia Y., Liu X., Zheng Y., et al., *Enteric bacterial protein AvrA promotes colonic tumorigenesis and activates colonic beta-catenin signaling pathway.* Oncogenesis, 2014. **3**(6): p. e105.
22. Scott N., Whittle E., Jeraldo P. and Chia N., *A systemic review of the role of enterotoxic Bacteroides fragilis in colorectal cancer.* Neoplasia, 2022. **29**: p. 100797.
23. Peuker K., Muff S., Wang J., Künzel S., Bosse E., Zeissig Y., et al., *Epithelial calcineurin controls microbiota-dependent intestinal tumor development.* Nat Med, 2016. **22**(5): p. 506-15.
24. Mu W., Jia Y., Chen X., Li H., Wang Z. and Cheng B., *Intracellular Porphyromonas gingivalis Promotes the Proliferation of Colorectal Cancer Cells via the MAPK/ERK Signaling Pathway.* Front Cell Infect Microbiol, 2020. **10**: p. 584798.
25. Chen Y., Huang Z., Tang Z., Huang Y., Huang M., Liu H., et al., *More Than Just a Periodontal Pathogen -the Research Progress on Fusobacterium nucleatum.* Front Cell Infect Microbiol, 2022. **12**: p. 815318.
26. Singh N., Gurav A., Sivaprakasam S., Brady E., Padia R., Shi H., et al., *Activation of Gpr109a, receptor for niacin and the commensal metabolite butyrate, suppresses colonic inflammation and carcinogenesis.* Immunity, 2014. **40**(1): p. 128-39.
27. Macia L., Nanan R., Hosseini-Beheshti E. and Grau G.E., *Host- and Microbiota-Derived Extracellular Vesicles, Immune Function, and Disease Development.* Int J Mol Sci, 2019. **21**(1).
28. Chen G., Huang A.C., Zhang W., Zhang G., Wu M., Xu W., et al., *Exosomal PD-L1 contributes to immunosuppression and is associated with anti-PD-1 response.* Nature, 2018. **560**(7718): p. 382-386.
29. Xu R., Rai A., Chen M., Suwakulsiri W., Greening D.W. and Simpson R.J., *Extracellular vesicles in cancer - implications for future improvements in cancer care.* Nat Rev Clin Oncol, 2018. **15**(10): p. 617-638.
30. Behzadi E., Mahmoodzadeh Hosseini H. and Imani Fooladi A.A., *The inhibitory impacts of Lactobacillus rhamnosus GG-derived extracellular vesicles on the growth of hepatic cancer cells.* Microb Pathog, 2017. **110**: p. 1-6.
31. Kim O.Y., Park H.T., Dinh N.T.H., Choi S.J., Lee J., Kim J.H., et al., *Bacterial outer membrane vesicles suppress tumor by interferon-γ-mediated antitumor response.* Nat Commun, 2017. **8**(1): p. 626.
32. Konishi H., Fujiya M., Tanaka H., Ueno N., Moriichi K., Sasajima J., et al., *Probiotic-derived ferrichrome inhibits colon cancer progression via JNK-mediated apoptosis.* Nat Commun, 2016. **7**: p. 12365.
33. Basak S. and Duttaroy A.K., *Conjugated Linoleic Acid and Its Beneficial Effects in Obesity, Cardiovascular Disease, and Cancer.* Nutrients, 2020. **12**(7).
34. Ma H., Yu Y., Wang M., Li Z., Xu H., Tian C., et al., *Correlation between microbes and colorectal cancer: tumor apoptosis is induced by sitosterols through promoting gut microbiota to produce short-chain fatty acids.* Apoptosis, 2019. **24**(1-2): p. 168-183.
35. Manilla V., Di Tommaso N., Santopaolo F., Gasbarrini A. and Ponziani F.R., *Endotoxemia and Gastrointestinal Cancers: Insight into the Mechanisms Underlying a Dangerous Relationship.* Microorganisms, 2023. **11**(2).
36. Shetab Boushehri M.A. and Lamprecht A., *TLR4-Based Immunotherapeutics in Cancer: A Review of the Achievements and Shortcomings.* Mol Pharm, 2018. **15**(11): p. 4777-4800.

37. Massart C., Poirier C., Fergelot P., Fardel O. and Gibassier J., *Effect of sodium butyrate on doxorubicin resistance and expression of multidrug resistance genes in thyroid carcinoma cells.* Anticancer Drugs, 2005. **16**(3): p. 255-61.
38. Dmitrieva-Posocco O., Wong A.C., Lundgren P., Golos A.M., Descamps H.C., Dohnalová L., et al., *β-Hydroxybutyrate suppresses colorectal cancer.* Nature, 2022. **605**(7908): p. 160-165.
39. Miyamoto J., Igarashi M., Watanabe K., Karaki S.I., Mukouyama H., Kishino S., et al., *Gut microbiota confers host resistance to obesity by metabolizing dietary polyunsaturated fatty acids.* Nat Commun, 2019. **10**(1): p. 4007.
40. Kaliannan K., Li X.Y., Wang B., Pan Q., Chen C.Y., Hao L., et al., *Multi-omic analysis in transgenic mice implicates omega-6/omega-3 fatty acid imbalance as a risk factor for chronic disease.* Commun Biol, 2019. **2**: p. 276.
41. Teame T., Wang A., Xie M., Zhang Z., Yang Y., Ding Q., et al., *Paraprobiotics and Postbiotics of Probiotic Lactobacilli, Their Positive Effects on the Host and Action Mechanisms: A Review.* Front Nutr, 2020. **7**: p. 570344.
42. den Hartigh L.J., *Conjugated Linoleic Acid Effects on Cancer, Obesity, and Atherosclerosis: A Review of Pre-Clinical and Human Trials with Current Perspectives.* Nutrients, 2019. **11**(2).
43. Baskar R., Lee K.A., Yeo R. and Yeoh K.W., *Cancer and radiation therapy: current advances and future directions.* Int J Med Sci, 2012. **9**(3): p. 193-9.
44. Shah C., Bauer-Nilsen K., McNulty R.H. and Vicini F., *Novel radiation therapy approaches for breast cancer treatment.* Semin Oncol, 2020. **47**(4): p. 209-216.
45. Johnson L.B., Riaz A.A., Adawi D., Wittgren L., Bäck S., Thornberg C., et al., *Radiation enteropathy and leucocyte-endothelial cell reactions in a refined small bowel model.* BMC Surg, 2004. **4**: p. 10.
46. Zhao Y., Zhang J., Han X. and Fan S., *Total body irradiation induced mouse small intestine senescence as a late effect.* J Radiat Res, 2019. **60**(4): p. 442-450.
47. Uribe-Herranz M., Rafail S., Beghi S., Gil-de-Gómez L., Verginadis I., Bittinger K., et al., *Gut microbiota modulate dendritic cell antigen presentation and radiotherapy-induced antitumor immune response.* J Clin Invest, 2020. **130**(1): p. 466-479.
48. Paulos C.M., Wrzesinski C., Kaiser A., Hinrichs C.S., Chieppa M., Cassard L., et al., *Microbial translocation augments the function of adoptively transferred self/tumor-specific CD8+ T cells via TLR4 signaling.* J Clin Invest, 2007. **117**(8): p. 2197-204.
49. Webb G.J., Brooke R. and De Silva A.N., *Chronic radiation enteritis and malnutrition.* J Dig Dis, 2013. **14**(7): p. 350-7.
50. Garczyk A., Kaliciak I., Drogowski K., Horwat P., Kopeć S., Staręga Z., et al., *Influence of Probiotics in Prevention and Treatment of Patients Who Undergo Chemotherapy or/and Radiotherapy and Suffer from Mucositis, Diarrhoea, Constipation, Nausea and Vomiting.* J Clin Med, 2022. **11**(12).
51. Peterson D.E., Boers-Doets C.B., Bensadoun R.J. and Herrstedt J., *Management of oral and gastrointestinal mucosal injury: ESMO Clinical Practice Guidelines for diagnosis, treatment, and follow-up.* Ann Oncol, 2015. **26 Suppl 5**: p. v139-51.
52. Yahyapour R., Motevaseli E., Rezaeyan A., Abdollahi H., Farhood B., Cheki M., et al., *Reduction-oxidation (redox) system in radiation-induced normal tissue injury: molecular mechanisms and implications in radiation therapeutics.* Clin Transl Oncol, 2018. **20**(8): p. 975-988.
53. Ciorba M.A., Riehl T.E., Rao M.S., Moon C., Ee X., Nava G.M., et al., *Lactobacillus probiotic protects intestinal epithelium from radiation injury in a TLR-2/cyclo-oxygenase-2-dependent manner.* Gut, 2012. **61**(6): p. 829-38.
54. Chitapanarux I., Chitapanarux T., Traisathit P., Kudumpee S., Tharavichitkul E. and Lorvidhaya V., *Randomized controlled trial of live lactobacillus acidophilus plus bifidobacterium bifidum in prophylaxis of diarrhea during radiotherapy in cervical cancer patients.* Radiat Oncol, 2010. **5**: p. 31.
55. Agirman G., Yu K.B. and Hsiao E.Y., *Signaling inflammation across the gut-brain axis.* Science, 2021. **374**(6571):

p. 1087-1092.
56. Sanders M.E., Guarner F., Guerrant R., Holt P.R., Quigley E.M., Sartor R.B., et al., *An update on the use and investigation of probiotics in health and disease.* Gut, 2013. **62**(5): p. 787-96.
57. Ahrén I.L., Bjurberg M., Steineck G., Bergmark K. and Jeppsson B., *Decreasing the Adverse Effects in Pelvic Radiation Therapy: A Randomized Controlled Trial Evaluating the Use of Probiotics.* Adv Radiat Oncol, 2023. **8**(1): p. 101089.
58. Crawford P.A. and Gordon J.I., *Microbial regulation of intestinal radiosensitivity.* Proc Natl Acad Sci U S A, 2005. **102**(37): p. 13254-9.
59. Iida N., Dzutsev A., Stewart C.A., Smith L., Bouladoux N., Weingarten R.A., et al., *Commensal bacteria control cancer response to therapy by modulating the tumor microenvironment.* Science, 2013. **342**(6161): p. 967-70.
60. Chu F.F., Esworthy R.S., Chu P.G., Longmate J.A., Huycke M.M., Wilczynski S., et al., *Bacteria-induced intestinal cancer in mice with disrupted Gpx1 and Gpx2 genes.* Cancer Res, 2004. **64**(3): p. 962-8.
61. Liu L., Bai Y., Xiang L., Qi W., Gao L., Li X., et al., *Interaction between gut microbiota and tumour chemotherapy.* Clin Transl Oncol, 2022. **24**(12): p. 2330-2341.
62. Wang L., Wang R., Wei G.Y., Wang S.M. and Du G.H., *Dihydrotanshinone attenuates chemotherapy-induced intestinal mucositis and alters fecal microbiota in mice.* Biomed Pharmacother, 2020. **128**: p. 110262.
63. Lian Q., Xu J., Yan S., Huang M., Ding H., Sun X., et al., *Chemotherapy-induced intestinal inflammatory responses are mediated by exosome secretion of double-strand DNA via AIM2 inflammasome activation.* Cell Res, 2017. **27**(6): p. 784-800.
64. Stringer A.M., *Interaction between host cells and microbes in chemotherapy-induced mucositis.* Nutrients, 2013. **5**(5): p. 1488-99.
65. Su J., Li D., Chen Q., Li M., Su L., Luo T., et al., *Anti-breast Cancer Enhancement of a Polysaccharide From Spore of Ganoderma lucidum With Paclitaxel: Suppression on Tumor Metabolism With Gut Microbiota Reshaping.* Front Microbiol, 2018. **9**: p. 3099.
66. Gui Q.F., Lu H.F., Zhang C.X., Xu Z.R. and Yang Y.H., *Well-balanced commensal microbiota contributes to anti-cancer response in a lung cancer mouse model.* Genet Mol Res, 2015. **14**(2): p. 5642-51.
67. Chattopadhyay I., Nandi D. and Nag A., *The pint- sized powerhouse: Illuminating the mighty role of the gut microbiome in improving the outcome of anti- cancer therapy.* Semin Cancer Biol, 2021. **70**: p. 98-111.
68. Viaud S., Saccheri F., Mignot G., Yamazaki T., Daillère R., Hannani D., et al., *The intestinal microbiota modulates the anticancer immune effects of cyclophosphamide.* Science, 2013. **342**(6161): p. 971-6.
69. Geller L.T. and Straussman R., *Intratumoral bacteria may elicit chemoresistance by metabolizing anticancer agents.* Mol Cell Oncol, 2018. **5**(1): p. e1405139.
70. Yazdi M.H., Mahdavi M., Setayesh N., Esfandyar M. and Shahverdi A.R., *Selenium nanoparticle-enriched Lactobacillus brevis causes more efficient immune responses in vivo and reduces the liver metastasis in metastatic form of mouse breast cancer.* Daru, 2013. **21**(1): p. 33.
71. Aragón F., Carino S., Perdigón G. and de Moreno de LeBlanc A., *The administration of milk fermented by the probiotic Lactobacillus casei CRL 431 exerts an immunomodulatory effect against a breast tumour in a mouse model.* Immunobiology, 2014. **219**(6): p. 457-64.
72. Lakritz J.R., Poutahidis T., Levkovich T., Varian B.J., Ibrahim Y.M., Chatzigiagkos A., et al., *Beneficial bacteria stimulate host immune cells to counteract dietary and genetic predisposition to mammary cancer in mice.* Int J Cancer, 2014. **135**(3): p. 529-40.
73. An J. and Ha E.M., *Combination Therapy of Lactobacillus plantarum Supernatant and 5-Fluouracil Increases Chemosensitivity in Colorectal Cancer Cells.* J Microbiol Biotechnol, 2016. **26**(8): p. 1490-503.
74. Geller L.T., Barzily-Rokni M., Danino T., Jonas O.H., Shental N., Nejman D., et al., *Potential role of intratumor*

bacteria in mediating tumor resistance to the chemotherapeutic drug gemcitabine. Science, 2017. **357**(6356): p. 1156-1160.

75. Yu T., Guo F., Yu Y., Sun T., Ma D., Han J., et al., *Fusobacterium nucleatum Promotes Chemoresistance to Colorectal Cancer by Modulating Autophagy.* Cell, 2017. **170**(3): p. 548-563.e16.
76. Yi Y., Shen L., Shi W., Xia F., Zhang H., Wang Y., et al., *Gut Microbiome Components Predict Response to Neoadjuvant Chemoradiotherapy in Patients with Locally Advanced Rectal Cancer: A Prospective, Longitudinal Study.* Clin Cancer Res, 2021. **27**(5): p. 1329-1340.
77. Gopalakrishnan V., Helmink B.A., Spencer C.N., Reuben A. and Wargo J.A., *The Influence of the Gut Microbiome on Cancer, Immunity, and Cancer Immunotherapy.* Cancer Cell, 2018. **33**(4): p. 570-580.
78. Sims T.T., El Alam M.B., Karpinets T.V., Dorta-Estremera S., Hegde V.L., Nookala S., et al., *Gut microbiome diversity is an independent predictor of survival in cervical cancer patients receiving chemoradiation.* Commun Biol, 2021. **4**(1): p. 237.
79. Mao J., Wang D., Long J., Yang X., Lin J., Song Y., et al., *Gut microbiome is associated with the clinical response to anti-PD-1 based immunotherapy in hepatobiliary cancers.* J Immunother Cancer, 2021. **9**(12).
80. Vétizou M., Pitt J.M., Daillère R., Lepage P., Waldschmitt N., Flament C., et al., *Anticancer immunotherapy by CTLA-4 blockade relies on the gut microbiota.* Science, 2015. **350**(6264): p. 1079-84.
81. Sivan A., Corrales L., Hubert N., Williams J.B., Aquino-Michaels K., Earley Z.M., et al., *Commensal Bifidobacterium promotes antitumor immunity and facilitates anti-PD-L1 efficacy.* Science, 2015. **350**(6264): p. 1084-9.
82. Routy B., Le Chatelier E., Derosa L., Duong C.P.M., Alou M.T., Daillère R., et al., *Gut microbiome influences efficacy of PD-1-based immunotherapy against epithelial tumors.* Science, 2018. **359**(6371): p. 91-97.
83. Zheng Y., Wang T., Tu X., Huang Y., Zhang H., Tan D., et al., *Gut microbiome affects the response to anti-PD-1 immunotherapy in patients with hepatocellular carcinoma.* J Immunother Cancer, 2019. **7**(1): p. 193.
84. Song P., Yang D., Wang H., Cui X., Si X., Zhang X., et al., *Relationship between intestinal flora structure and metabolite analysis and immunotherapy efficacy in Chinese NSCLC patients.* Thorac Cancer, 2020. **11**(6): p. 1621-1632.
85. Lalani A.A., Xie W., Braun D.A., Kaymakcalan M., Bossé D., Steinharter J.A., et al., *Effect of Antibiotic Use on Outcomes with Systemic Therapies in Metastatic Renal Cell Carcinoma.* Eur Urol Oncol, 2020. **3**(3): p. 372-381.
86. Pinato D.J., Howlett S., Ottaviani D., Urus H., Patel A., Mineo T., et al., *Association of Prior Antibiotic Treatment With Survival and Response to Immune Checkpoint Inhibitor Therapy in Patients With Cancer.* JAMA Oncol, 2019. **5**(12): p. 1774-1778.
87. Elkrief A., El Raichani L., Richard C., Messaoudene M., Belkaid W., Malo J., et al., *Antibiotics are associated with decreased progression-free survival of advanced melanoma patients treated with immune checkpoint inhibitors.* Oncoimmunology, 2019. **8**(4): p. e1568812.
88. Zhao S., Gao G., Li W., Li X., Zhao C., Jiang T., et al., *Antibiotics are associated with attenuated efficacy of anti-PD-1/PD-L1 therapies in Chinese patients with advanced non-small cell lung cancer.* Lung Cancer, 2019. **130**: p. 10-17.
89. Tinsley N., Zhou C., Tan G., Rack S., Lorigan P., Blackhall F., et al., *Cumulative Antibiotic Use Significantly Decreases Efficacy of Checkpoint Inhibitors in Patients with Advanced Cancer.* Oncologist, 2020. **25**(1): p. 55-63.
90. Wilson B.E., Routy B., Nagrial A. and Chin V.T., *The effect of antibiotics on clinical outcomes in immune-checkpoint blockade: a systematic review and meta-analysis of observational studies.* Cancer Immunol Immunother, 2020. **69**(3): p. 343-354.
91. Nie D., Fang Q., Cheng J., Li B., Li M., Wang H., et al., *The intestinal flora of patients with GHPA affects the growth and the expression of PD-L1 of tumor.* Cancer Immunol Immunother, 2022. **71**(5): p. 1233-1245.

92. Mohiuddin J.J., Chu B., Facciabene A., Poirier K., Wang X., Doucette A., et al., *Association of Antibiotic Exposure With Survival and Toxicity in Patients With Melanoma Receiving Immunotherapy.* J Natl Cancer Inst, 2021. **113**(2): p. 162-170.
93. Chalabi M., Cardona A., Nagarkar D.R., Dhawahir Scala A., Gandara D.R., Rittmeyer A., et al., *Efficacy of chemotherapy and atezolizumab in patients with non-small-cell lung cancer receiving antibiotics and proton pump inhibitors: pooled post hoc analyses of the OAK and POPLAR trials.* Ann Oncol, 2020. **31**(4): p. 525-531.
94. Derosa L., Hellmann M.D., Spaziano M., Halpenny D., Fidelle M., Rizvi H., et al., *Negative association of antibiotics on clinical activity of immune checkpoint inhibitors in patients with advanced renal cell and non-small-cell lung cancer.* Ann Oncol, 2018. **29**(6): p. 1437-1444.
95. Hopkins A.M., Kichenadasse G., Karapetis C.S., Rowland A. and Sorich M.J., *Concomitant Antibiotic Use and Survival in Urothelial Carcinoma Treated with Atezolizumab.* Eur Urol, 2020. **78**(4): p. 540-543.
96. Kim H., Lee J.E., Hong S.H., Lee M.A., Kang J.H. and Kim I.H., *The effect of antibiotics on the clinical outcomes of patients with solid cancers undergoing immune checkpoint inhibitor treatment: a retrospective study.* BMC Cancer, 2019. **19**(1): p. 1100.
97. Ouaknine Krief J., Helly de Tauriers P., Dumenil C., Neveux N., Dumoulin J., Giraud V., et al., *Role of antibiotic use, plasma citrulline and blood microbiome in advanced non-small cell lung cancer patients treated with nivolumab.* J Immunother Cancer, 2019. **7**(1): p. 176.
98. Cheng W.Y., Wu C.Y. and Yu J., *The role of gut microbiota in cancer treatment: friend or foe?* Gut, 2020. **69**(10): p. 1867-1876.
99. Gopalakrishnan V., Spencer C.N., Nezi L., Reuben A., Andrews M.C., Karpinets T.V., et al., *Gut microbiome modulates response to anti-PD-1 immunotherapy in melanoma patients.* Science, 2018. **359**(6371): p. 97-103.
100. Mekadim C., Skalnikova H.K., Cizkova J., Cizkova V., Palanova A., Horak V., et al., *Dysbiosis of skin microbiome and gut microbiome in melanoma progression.* BMC Microbiol, 2022. **22**(1): p. 63.
101. Larkin J., Hodi F.S. and Wolchok J.D., *Combined Nivolumab and Ipilimumab or Monotherapy in Untreated Melanoma.* N Engl J Med, 2015. **373**(13): p. 1270-1.
102. Chaput N., Lepage P., Coutzac C., Soularue E., Le Roux K., Monot C., et al., *Baseline gut microbiota predicts clinical response and colitis in metastatic melanoma patients treated with ipilimumab.* Ann Oncol, 2017. **28**(6): p. 1368-1379.
103. Dubin K., Callahan M.K., Ren B., Khanin R., Viale A., Ling L., et al., *Intestinal microbiome analyses identify melanoma patients at risk for checkpoint-blockade-induced colitis.* Nat Commun, 2016. **7**: p. 10391.
104. Shi Y., Zheng W., Yang K., Harris K.G., Ni K., Xue L., et al., *Intratumoral accumulation of gut microbiota facilitates CD47-based immunotherapy via STING signaling.* J Exp Med, 2020. **217**(5).
105. Chao M.P., Alizadeh A.A., Tang C., Myklebust J.H., Varghese B., Gill S., et al., *Anti-CD47 antibody synergizes with rituximab to promote phagocytosis and eradicate non-Hodgkin lymphoma.* Cell, 2010. **142**(5): p. 699-713.
106. Zhang W., Huang Q., Xiao W., Zhao Y., Pi J., Xu H., et al., *Advances in Anti-Tumor Treatments Targeting the CD47/SIRPα Axis.* Front Immunol, 2020. **11**: p. 18.
